# Broad-Scale Recombination Patterns Underlying Proper Disjunction in Humans

**DOI:** 10.1371/journal.pgen.1000658

**Published:** 2009-09-18

**Authors:** Adi Fledel-Alon, Daniel J. Wilson, Karl Broman, Xiaoquan Wen, Carole Ober, Graham Coop, Molly Przeworski

**Affiliations:** 1 Department of Human Genetics, University of Chicago, Chicago, Illinois, United States of America; 2 Department of Biostatistics and Medical Informatics, University of Wisconsin, Madison, Wisconsin, United States of America; 3 Department of Statistics, University of Chicago, Chicago, Illinois, United States of America; 4 Evolution and Ecology Section, University of California Davis, Davis, California, United States of America; 5 Department of Ecology and Evolution, University of Chicago, Chicago, Illinois, United States of America; The University of North Carolina at Chapel Hill, United States of America

## Abstract

Although recombination is essential to the successful completion of human meiosis, it remains unclear how tightly the process is regulated and over what scale. To assess the nature and stringency of constraints on human recombination, we examined crossover patterns in transmissions to viable, non-trisomic offspring, using dense genotyping data collected in a large set of pedigrees. Our analysis supports a requirement for one chiasma per chromosome rather than per arm to ensure proper disjunction, with additional chiasmata occurring in proportion to physical length. The requirement is not absolute, however, as chromosome 21 seems to be frequently transmitted properly in the absence of a chiasma in females, a finding that raises the possibility of a back-up mechanism aiding in its correct segregation. We also found a set of double crossovers in surprisingly close proximity, as expected from a second pathway that is not subject to crossover interference. These findings point to multiple mechanisms that shape the distribution of crossovers, influencing proper disjunction in humans.

## Author Summary

In humans, as in most sexually reproducing organisms, recombination plays a fundamental role in meiosis, helping to align chromosomes and to ensure their proper segregation. Recombination events are tightly regulated both in terms of their minimum number (the rule of “crossover assurance”) and placement (due to “crossover interference”). Accumulating evidence, however, suggests that recombination patterns are highly variable among humans, raising numerous questions about the nature and stringency of crossover assurance and interference. We took a first step towards answering these questions by examining patterns of recombination in gametes inherited by viable, non-trisomic offspring. We found that the minimum number of crossovers is tightly regulated at the level of a chromosome (rather than chromosome arm), but with a notable exception: in females, chromosome 21 appears to frequently segregate properly in the absence of a crossover. We also found a set of double recombination events in surprisingly close proximity, consistent with a pathway not subject to crossover interference. These findings suggest that there are multiple mechanisms of recombination in human meiosis, which may buffer the effects of inter-individual variation in rates.

## Introduction

Like most sexually reproducing organisms, humans undergo meiotic recombination. This process plays an important role in evolutionary dynamics, generating new combination of alleles on which natural selection can act ([1] and references therein), and in DNA repair. In humans, recombination is also fundamental to the successful completion of meiosis, helping to align homologous chromosomes and to ensure their proper disjunction [2]. Too little recombination or an abnormal placement of crossovers along the genome often results in aneuploidy, an outcome that leads to fetal loss or to severe developmental disabilities [3]. Thus, errors in the recombination process are clearly highly deleterious.

Nonetheless, there is substantial variation in recombination rates and patterns among humans. Individuals differ in their mean number of crossovers across the genome [4]–[6], in part due to genetic variation [7]. They also differ in the intensity of individual recombination hotspots, and in their use of hotspots genome-wide, variability that is again at least partly heritable [8],[9]. Moreover, this variation has detectable fitness consequences: mothers with a higher mean recombination rate have (slightly) more viable offspring [5],[9]. Together, these findings suggest that human recombination is naturally viewed as a quantitative phenotype subject to selection.

This perspective raises a number of questions about the selective pressures on recombination due to its role in meiosis, notably about their nature and stringency and the extent to which the system is buffered against variation. Answers to these questions have important implications for the relationship between recombination rate variation and the susceptibility to non-disjunction. Such answers are also essential to the study of the evolution of recombination rates and of genome dynamics [10],[11].

Chiasmata, the physical connections between chromatids later processed into crossovers, help to bind the homologs together, thereby aiding in their proper disjunction. In the absence of a chiasma, segregation is thought to be haphazard and to frequently result in aneuploidy, a role for recombination that likely imposes strong selective pressures for at least one chiasma per chromosome, even when the mean number is close to 1 [12].

In most species, however, the total number of chiasmata (in males and females) far exceeds the number of chromosomes (cf. [10]). Foci counts of MLH1, a mismatch repair protein that serves as markers for most (but perhaps not all) human crossovers, indicate that there is a chiasma on each chromosome arm examined [13]. Moreover, across mammals, the number of chromosome arms (i.e., two for metacentric chromosomes) appears to be a better predictor of the mean number of crossovers than is the number of chromosomes [10],[14]. These cytogenetic and evolutionary lines of evidence have led to the suggestion that, with rare exceptions, one chiasma per chromosome *arm* is required for proper disjunction ([15] and references therein; see also [10],[13],[14],[16],[17]).

The role of recombination in human meiosis shapes the placement of crossovers as well as their number. When more than one crossing-over event occurs on the same chromosome, the events are not spaced randomly, but instead tend to occur farther apart than expected by chance. The more even spacing of events on chromosomes due to this “crossover interference” may serve to further reduce the risk of non-disjunction [18]. While several models of interference have been proposed, the phenomenon remains poorly understood. In particular, evidence from model organisms, including mice, suggests that a small subset of events result from a second pathway, not subject to crossover interference [16]. A central prediction of the two-pathway hypothesis is the presence of rare, double recombinants in close proximity (see [19] for details).

Here, we used human pedigree data to assess whether the number of chiasmata is tightly regulated at the level of chromosomes or chromosome arms and to examine how often proper disjunction occurs in the absence of a chiasma. We also took advantage of the high spatial resolution of the data to investigate the spacing of crossover events.

## Results

Our point of departure was the number of crossovers observed in transmissions to viable, non-trisomic offspring (Supplementary Table 1 in Text S1). We constructed this distribution from 576 meioses in a large European-American pedigree (see Methods). The families were genotyped at approximately 400,000 single nucleotide polymorphisms (SNPs), providing excellent coverage of the genome (see Supplementary Table 2 in Text S1) and allowing us to localize crossover locations with high resolution [9]. For chromosome 21, we also considered the number of crossovers in an additional 152 female transmissions; these were inferred in European-American families that had been typed for 133 SNPs [20]. The distributions do not differ significantly between the two data sets (p = 0.86 by a Fisher's Exact Test).

The crossovers observed in gametes cannot be equated to the number of chiasmata in tetrads since, for every chiasma, only two of the four chromatids are recombinants. (The term chiasma is sometimes reserved for the physical manifestation of a crossover detected in cytogenetic studies; here, we employ it to denote a crossover in the tetrad, and use the term crossover to refer specifically to genetic exchanges visible in transmitted gametes.) We used the observed distribution of the number of crossovers in gametes to infer the distribution of chiasmata in the tetrads (also called bivalents) [21]. We were particularly interested in assessing how often there was no chiasma in the tetrad, i.e., how often nullichiasmatic chromosomes segregated properly. Following previous studies (e.g., [22],[23]), we assumed that one of the four chromatids is transmitted at random. We further assumed that there is no chromatid interference, i.e., that when there is more than one chiasma in the tetrad, the pair of chromatids involved in each genetic exchange is chosen independently and at random (cf. [24]). Under this model, given a chiasma, there is always a probability of one half of transmitting a recombinant chromatid. Thus, if there are one or more chiasmata per tetrad, at most half the gametes are expected to be non-recombinants. Assuming that no crossovers are missed due to insufficient marker coverage, a significant excess of non-recombinant gametes beyond one-half provides unequivocal evidence for the existence of tetrads that segregate properly without a chiasma. More generally, the shape of the observed distribution of crossover counts is informative about underlying patterns of recombination in tetrads.

To infer the distribution of the number of chiasmata in tetrads, we used two approaches: a likelihood method, variants of which have been applied in a number of studies [20],[22],[23],[25],[26], as well as a Bayesian approach that we developed and which we believe presents a number of advantages (see Methods). Results from the two methods were similar (see Supplementary Figure 1 in Text S1); to facilitate the comparison to earlier studies, we present those obtained from the likelihood approach.

**Figure 1:**
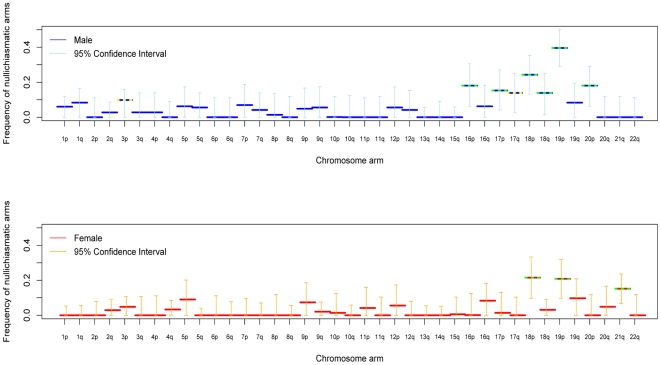
For each chromosomal arm, the estimated fraction of bivalents that segregated properly without a chiasma, in male (blue) and female (red) transmissions. Shown are maximum likelihood estimates and 95% confidence intervals (see Methods). Estimates significantly above 0 at the 5% level are indicated by a yellow dotted line and at the 1% level by a green dotted line. The data for chromosome 21 combine two sets of pedigrees (see Methods).

Applying the approach to crossover data for each chromosomal arm separately, we found overwhelming evidence for frequent, proper disjunction in the absence of a chiasma (Figure 1). In both male and female transmissions, estimates of nullichiasmatic arms are as high as 20–40% for the smaller metacentric chromosomes, but are significantly above 0 even for some of the larger chromosome arms. Given the dense marker coverage of each arm (see Supplementary Table 2 in Text S1), we are missing at most a small fraction of crossover events, rendering our estimates robust. Thus, our findings establish that, in humans, proper segregation does not require the tight regulation of the number of chiasmata per chromosome arm.

**Figure 2:**
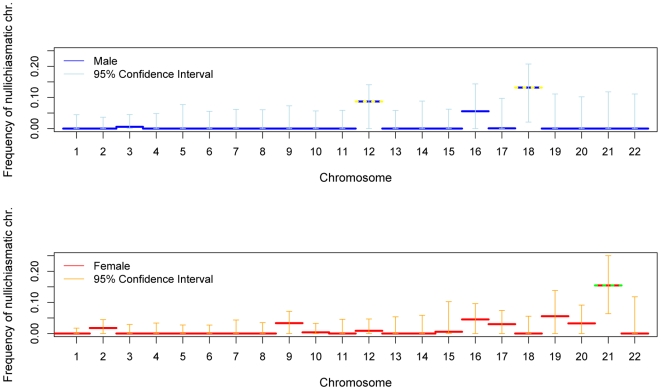
For each chromosome, the estimated fraction of bivalents that segregated properly without a chiasma, in male (blue) and female (red). See the legend of Figure 1 for additional details.

**Figure 3:**
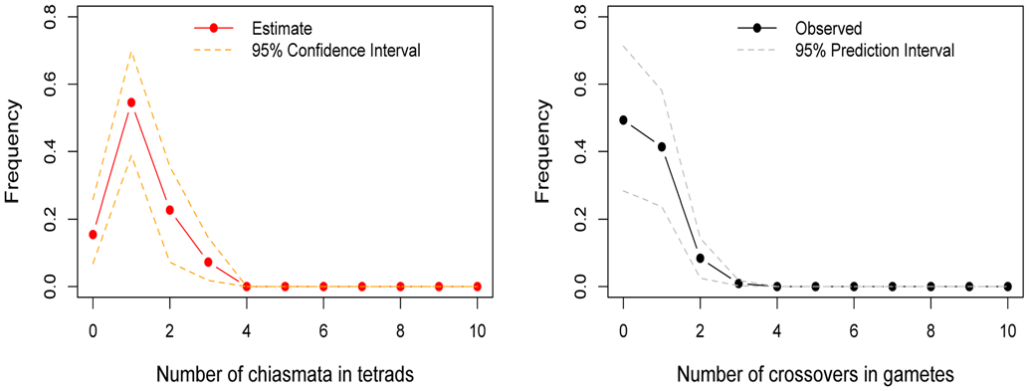
The estimated distribution of chiasmata per bivalent, in female transmissions of chromosome 21. To the left is the inferred distribution of chiasmata per bivalent, obtained from a maximum-likelihood method. The point estimates are shown as filled red circles and the 95% confidence intervals as orange dotted lines (see Methods). To the right is the observed distribution of crossovers among female transmissions to viable, non-trisomic offspring, in two sets of pedigrees (see Methods). As a test of the goodness of fit of the model, we also show (in gray dotted lines) the prediction intervals obtained from 5000 simulations where we binomially sample the number of crossovers per gamete distribution given the estimated distribution of chiasmata per bivalent; as can be seen, the data fit well within the 95% prediction intervals (see Methods). The data indicate that a substantial and significant (p = 0.0002) fraction of female transmissions involved a nullichiasmatic chromosome 21.

We also assessed the stringency of the rule at the level of the entire chromosome. With these data, we can only test whether nullichiasmatic bivalents *can* segregate properly for the smaller chromosomes, because for the larger chromosomes, they rarely occur (as the recombination rate is simply too high). Indeed, simulations suggest that, even if nullichiasmatic chromosomes always segregated properly, we would have high power to detect their transmission only for the eight smallest chromosomes in males and for chromosomes 21 and 22 in females (see Supplementary Methods in Text S1). As expected, the larger chromosomes show no evidence of nullichiasmatic transmissions to viable offspring (Figure 2). But among the smaller chromosomes, there are apparent exceptions. In males, we found evidence for proper segregation in the absence of a chiasma for two chromosomes: in over 0.1% (the lower 5%-tile) of cases for chromosome 12 (p = 0.0176), and in over 4.2% of cases for chromosome 18 (p = 0.0120). In females, in turn, we inferred that at least 7.3% (p = 0.0002) of chromosome 21 transmissions occur properly in the absence of a chiasma (Figure 3).

Given that the estimated fractions of nullichiasmatic bivalents are relatively small, they may be sensitive to a modest number of missing crossover events. To evaluate this possibility, we performed a number of checks. For chromosomes 12 and 18, these analyses suggested that our results are tentative, as a subset of families are missing informative markers for some of the telomeric regions—enough to potentially inflate the apparent number of non-recombinant gametes and lead to an over-estimate of the fraction of nullichiasmatic tetrads (see Supplementary Methods in Text S1). For chromosome 21, however, the two data sets that we analyzed have good marker coverage and are missing at most a small number of events, indicating that our results are reliable (see Supplementary Methods in Text S1).

In interpreting the chromosome 21 results, a second consideration is the number of tests performed. As noted above, for the larger chromosomes, the probability of the data will be very similar under a model allowing for nullichiasmatic chromosomes and one that does not; only for the small chromosomes could the p-value derived from our likelihood ratio test be small. This reasoning suggests that we should be correcting for many fewer than 22 tests—possibly as few as two. But even if we were to conservatively correct for 22 tests, the results for chromosome 21 remain significant (e.g., if we use a Bonferroni correction, p = 0.0044). Thus, in females at least, the requirement for one chiasma per chromosome is not absolute.

To learn more about patterns of recombination underlying proper disjunction in humans, we used the high resolution of crossover locations in our data in order to better characterize crossover interference. Overall estimates of the strength of crossover interference are similar to those previously reported based on a smaller data set [27], although potential differences emerge between sexes and among chromosomes (see Supplementary Figure 2 in Text S1). Also consistent with earlier studies [27],[28], we find that the centromere is not a barrier to interference (see Supplementary Figure 3 in Text S1). This suggests that when a chiasma occurs close to the centromere of one arm, it will increase the odds of the other arm being nullichiasmatic.

**Figure 4:**
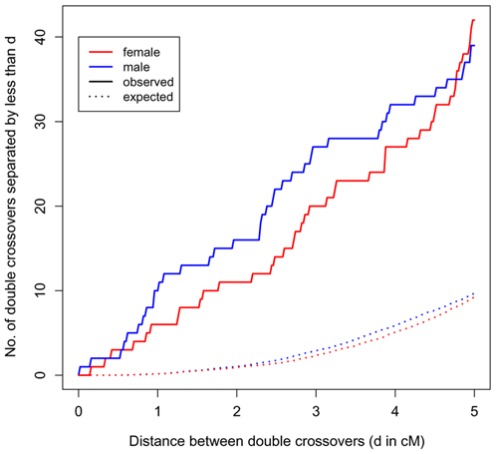
Double crossovers in close proximity. The number of double crossovers within 5 cM or less *versus* the number expected under a gamma renewal model, as estimated from the data (distances are based on sex-specific genetic maps; see Supplementary Methods in Text S1). In red are events in female transmissions; in blue are events in male transmissions. A version of this figure with stringently vetted double crossovers is presented in Supplementary Figure 4 in Text S1.

Because of the high density of our markers, we were able to identify a set of double crossovers in close proximity (<5 cM), distributed across families and genomic locations (see Supplementary Table 4 in Text S1). This observation is highly unexpected under the standard statistical model of interference, the gamma renewal model (Figure 4). The excess of tight double crossovers is still apparent even when we focus on a set of stringently vetted double crossovers, which likely under-estimates the true number (Supplementary Figure 4 in Text S1). Thus, in humans, a non-negligible number of double crossovers occur surprisingly close together.

## Discussion

**Figure 5:**
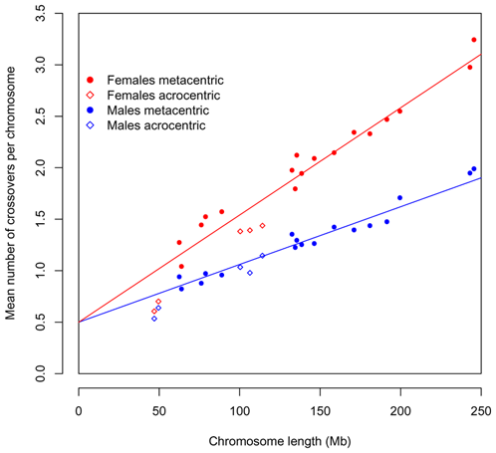
The total recombination rate per chromosome, in males and females, as predicted by an obligate chiasma per bivalent (i.e, an intercept at 0.5 crossovers per gamete) and the physical length of the chromosome. In red are the observed values for females and in blue the ones for males; circles denote metacentric chromosomes and diamonds acrocentric ones. The regression line is shown for each sex separately. In both cases, there is an excellent fit of this simple model to the data (R^2^ = 0.94 for females; R^2^ = 0.95 for males).

Our statistical analysis indicates that human crossovers are not regulated on the scale of an arm but on that of an entire chromosome (as found also in another mammal, shrews, using a cytogenetic approach [29]). In fact, the genetic length of a chromosome is extremely well predicted by a model that incorporates only two features: the need for one event and the length of the chromosome [19],[30] (see also Figure 5). The tight fit of the model further suggests that there are few chromosome-specific factors affecting the total recombination rate per chromosome and that beyond one event, additional crossovers occur in rough proportion to physical length.

As known for over half a century, the placement of these additional events is subject to positive crossover interference. Interestingly, however, this may not be true of all crossovers (see [19] for discussion). Indeed, we find an excess of rare, double crossovers in close proximity, which are better fit by the model of Housworth and Stahl (2003) [19], where there are two types of crossovers, only one of which is subject to interference, than by the standard interference model (see Supplementary Methods in Text S1). Thus, while other explanations remain, our results are consistent with the existence of a second crossover pathway in humans.

We also see exceptions to the rule of one chiasma per chromosome, most clearly for female (but not male; see Figure 2) transmissions of chromosome 21. Our analysis relies on assumptions of no chromatid interference and no meiotic drive of chromatids [31]. Violations of these assumptions could increase the variance in the transmission of non-recombinant chromatids, potentially serving as an alternative explanation for our findings. We extended our model to mimic this effect (see Supplementary Methods in Text S1) and found that a large variance in transmission probability could account for the observed distribution of crossovers on chromosome 21 even if there were an obligate chiasma to ensure proper disjunction (Supplementary Figure 5 in Text S1). However, studies in yeast, where this hypothesis can be tested directly, found no evidence of chromatid interference [24],[32]. In turn, if meiotic drive explains our findings, we might expect to see over-transmission of certain genotypes in females. Yet there is no evidence for transmission distortion of markers on chromosome 21 (see Supplementary Methods in Text S1). Additional explanations for our finding are strong, female-specific selection against recombinant gametes or the preferential transmission of non-recombinant chromatids. While both remain formal possibilities, of interest in their own right, there is no evidence for such selection in humans, nor is there (to our knowledge) a clear mechanism that would lead to the over transmission of non-recombinant chromatids.

Given that the model assumptions are well supported by studies in model organisms, and the frequent observation of trisomy 21 in humans [3], the most plausible interpretation of our findings is that nullichiasmatic chromosomes 21 occasionally experience proper disjunction. This conclusion is in qualitative agreement with the results of a subset of cytogenetic studies, which find that shorter bivalents sometimes lack an MLH1 focus (e.g., [33]–[35]); for example, Tease et al. [35] found that ∼3.5% of ooyctes from one female lacked a MLH1 focus on the chromosome 21 bivalent. However, the results of such studies vary markedly, likely due to the small number of cells and individuals that can be considered (e.g., [28], [34], [36]–[38]). Moreover, if there is a second crossover pathway in humans, MLH1 foci may not mark all crossovers [16]. Perhaps most importantly, in studies of pachytene cells, the fate of the daughter cells remains unknown [33]; in contrast, we are ascertaining viable offspring, where proper disjunction has clearly occurred.

Intriguingly, the pedigree data suggest that the proper segregation of nullichiasmatic chromosome 21 is fairly frequent. In the absence of a back-up mechanism to aid in the proper disjunction of nullichiasmatic chromosomes, chromatids are expected to segregate randomly to the poles, resulting in chromosomally unbalanced daughter cells in half the outcomes and in balanced cells in the other half. Thus, the rate of proper segregation of nullichiasmatic chromosomes should equal the rate of aneuploidy (in fact, it should be quite a bit lower, since aneuploidy has other sources). Yet fewer than 1% of all conceptions are thought to be aneuploid for chromosome 21—an estimate markedly lower than the fraction of nullichiasmatic transmissions that seem to segregate properly. This large discrepancy raises the possibility of a back-up mechanism in humans, similar to those that exist in Drosophila and yeast [33].

The absence of a chiasma on maternal chromosome 21 is known to be a risk factor for trisomy 21—the main cause of Down's syndrome [20],[22],[39]. If there is a back-up mechanism that aids in the proper disjunction of nullichiasmatic chromosomes, variation in its effectiveness (either across females or with age) could contribute to the risk of forming aneuploid gametes [31].

## Methods

### The observed distribution of the number of crossovers

We previously estimated the location of crossover events in a large Hutterite (a founder population of European origin) pedigree that had been genotyped using Affymetrix 500 K genotyping arrays [9]. To this end, we required K = 5 or more consecutive informative markers to call a crossover event [9]. At the scale of a megabase or more, our sex-specific genetic maps were highly concordant with the those of Kong et al. [40], which are based on more meioses but fewer markers [9].

For these analyses, we focused on 52 overlapping, nuclear families of four or more genotyped offspring, as simulations suggested that our algorithm is highly reliable for families of more than three children (for K = 5) [9]. Based on the total 576 meioses, we constructed the distribution of the number of crossovers in (male or female) gametes, for each chromosome. For female transmissions of chromosome 21, we supplemented our data set with crossover numbers reported in Oliver et al. (2008) [20]. All transmissions were to viable, non-trisomic offspring.

To assign crossovers to chromosome arms, we relied on the centromere gap location in build May 2004 of the human genome (as provided by http://genome.ucsc.edu/). Events were assigned to the p arm if the start of the interval within which they were localized was left of the centromere; remaining events were assigned to the q arm. When the intervals spanned the centromere boundary, we conservatively added the events to both arms; few events fell in this category (see Supplementary Table 1 in Text S1).

### Estimating the number of chiasmata in bivalents

Our starting point for inference was the model of Ott (1996) [25], in which the binomial distribution with parameter 0.5 describes the number of crossovers, *Y*, in a gamete given *X* chiasmata in the bivalent:
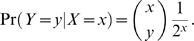
(1)


This model assumes no chromatid interference in the distribution of chiasmata across chromatid pairs; in the Supplementary Methods in Text S1, we describe a simple extension that mimics some of the effects of chromatid interference. The object of inference was the probability distribution of the number of chiasmata in bivalents, described by the vector *p*, where
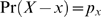
(2)is the probability of *x* chiasmata in a bivalent. For computational convenience, we assumed that *x* lies in a finite range 0…*x*_max_; we chose *x*_max_ = 20. To obtain maximum likelihood estimates of *p* for each chromosome and chromosome arm, we employed the EM algorithm of [25]. Confidence intervals were estimated using the parametric bootstrap procedure described in Yu and Feingold (2001) [23], based on 5000 permutations for chromosomes and 1000 for chromosome arms.

### Testing for the proper disjunction of nullichiasmatic bivalents

To test for the presence of nullichiasmatic bivalents among our sample of gametes (that necessarily underwent proper disjunction), we conducted a likelihood ratio test (LRT) of the unconstrained model (in which *p*_0_≥0) to the constrained model (*p*_0_ = 0). To fit the latter, we utilized the same EM algorithm subject to the additional constraint that *p*_0_ = 0. Because the asymptotic distribution for the LRT statistic is known to provide a conservative test [23], we calculated p-values using parametric bootstrap (again with 5000 permutations for chromosomes and 1000 for chromosome arms).

### A Bayesian approach to the problem

By taking a complementary, Bayesian approach to inference, we were able to incorporate extensive prior information about recombination patterns in order to improve inferential power, to quantify the deviation of *p* from a naïve model with no chromosome interference and to avoid possible statistical problems arising from a high-dimensional parameter and modest sample size.

Specifically, using the Kong et al. (2002) [40] estimate of the expected number of crossovers, *λ*, in a particular chromosome or chromosome arm, we employed a Dirichlet prior on *p* with parameter vector *α*, where
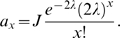
(3)


The prior expectation of *p_x_* is *α_x_*/*J*, which equals the Poisson probability of *x* chiasmata (given a mean of 2*λ*) and arises from a model of no chromosome interference. *J* controls the weight of prior information, and allows for deviation from this simple model. We explored values of *J* = 0.1, 1 and 5. Results were similar across values of *J* (not shown); for ease of interpretation, we present the results for *J* = 1. The Bayesian model was fit using Markov chain Monte Carlo (MCMC), which is described fully in the Supplementary Methods in Text S1.

### Characterizing the strength and nature of crossover interference

The gamma model, a standard model for crossover interference, was previously found to be a good fit to inter-crossover distances in humans and in a number of other organisms (e.g., [27],[41]). In the gamma model, the locations of the chiasmata on tetrads occur according to a stationary gamma renewal process, where the genetic distances between chiasmata follow a gamma distribution with shape and rate parameters ν and 2ν, respectively. Under the assumption of no chromatid interference, the locations of the crossovers are obtained by thinning the chiasma locations independently, with a probability of 1/2.

Following Broman and Weber (2000) [27], we estimated the parameter ν from our data, for each sex and each chromosome separately (Supplementary Figure 3 in Text S1). The value of ν estimated from the Hutterite data is similar to that obtained by [27], but with tighter confidence intervals due to the larger number of meioses available in the Hutterites. We find some evidence for variation between chromosomes and sexes in the strength of interference (i.e., variation in ν). However, given the lack of fit of the gamma model (Figure 4), these findings should be interpreted with caution.

## Supporting Information

Text S1 Supplementary methods, figures, and tables.
(1.14 MB PDF)
