# Supplementary material for: Broad-Scale Recombination Patterns Underlying Proper Disjunction in Humans
Source: PLoS Genet. 2009 Sep 18;5(9):e1000658. doi: 10.1371/journal.pgen.1000658 (PMC2734982; doi:10.1371/journal.pgen.1000658)

Supplementary Material for

Broad-scale recombination patterns underlying proper disjunction in humans

By Adi Fledel-Alon, Daniel J. Wilson, Karl Broman, Xiaoquan Wen, Carole Ober, Graham Coop and Molly Przeworski

Supplementary Materials, including:

p. 2 Power to detect the transmission of nullichiasmatic chromosomes
p. 3 The robustness of our results for chromosome arms
p. 4 The robustness of our results for chromosomes
p. 6 Characterizing crossover interference
p. 8 Vetting double crossovers in close proximity
p. 8 A two pathway model for crossover interference
p. 10 Modeling the effects of chromatid interference
p. 11 Testing for transmission distortion of genotypes on chromosome 21
p. 12 A Bayesian approach
p. 14 Model comparison using Bayes factors
p. 15 A maternal age effect on recombination patterns?
p. 16 References

Supplementary Tables and Figures, including:

p. 17 Table 1: The observed distribution of the number of crossovers per chromosome or chromosome arm
p. 21 Table 2: Marker coverage per chromosome or chromosome arm
p. 24 Table 3: List of stringently vetted double crossovers within 5 cM.
p. 26 Figure 1: Bayesian estimates of the fraction of nullichiasmatic bivalents and Bayes Factors.
p. 29 Figure 2: Sex-specific estimates of the parameter ν from the gamma model of chromosome interference.
p. 30 Figure 3: Correlation in the location of recombination events that occur on either side of the centromere.
p. 31 Figure 4: An excess of double crossovers in close proximity
p. 32 Figure 5: A model of chromatid interference and an obligate crossing-over per chromosome, for female chromosome 21.

Supplementary Methods Section.

Power to detect the transmission of nullichiasmatic chromosomes.

Our goal was to evaluate whether, when nullichiasmatic bivalents occur, their rate of proper disjunction is significantly greater than 0. For the larger chromosomes, we suspected that we would have little ability to answer this question, because the recombination rate is high enough that there is almost always a chiasma. To assess our power to detect the proper segregation of nullichiasmatic bivalents using the Hutterite data (see Methods in main text), we ran simulations for the different chromosomes. To this end, we considered two models: (i) A null model in which there is an obligate crossover per bivalent and the distribution of crossovers is otherwise specified by a Poisson distribution, with parameter the expected recombination rate of that chromosome (i.e., the "truncated Poisson model" [1]). (ii) An alternative model in which nullichiasmatic chromosomes are allowed and their frequency is specified by the same Poisson distribution. Both models assume that there is no crossover interference; model (ii) assumes that all nullichiasmatic chromosomes segregate properly. The first assumption is incorrect, but may nonetheless be a reasonable first approximation for the expected number of nullichiasmatic chromosomes. The second assumption leads to the best-case scenario for detecting the successful transmission of nullichiasmatic bivalents.

To generate "observed counts" for each chromosome, we used binomial sampling to draw from the underlying distribution of chiasmata under the alternative model (the use of binomial sampling mimics the sampling of recombinant chromatids). We then ran our likelihood method on the simulated data and assessed how often (out of 100 simulations) we obtained a significant likelihood ratio test at the 5% level (by the same procedure as on the actual data). We did so separately for males and females, as the two sexes are known to differ in many aspects of meiosis, including in their genetic map length and possibly in the stringency of meiotic checkpoints [2,3].

Chr	Males	Females
1	0.02	0.02
2	0.01	0
3	0.04	0.02
4	0.17	0.04
5	0.17	0.01
6	0.23	0.02
7	0.24	0.02
8	0.32	0.03
9	0.3	0
10	0.29	0.01
11	0.3	0.07
12	0.38	0.02
13	0.52	0.13
14	0.72	0.23
15	0.58	0.2

16	0.7	0.12
17	0.72	0.12
18	0.85	0.13
19	0.93	0.56
20	0.8	0.34
21	1	1
22	1	1

As can be seen, we only have substantial power (where substantial is arbitrarily defined as greater than 0.67) for chromosomes 21 and 22 in females, and the 8 smallest chromosomes in males. (We have power for more chromosomes in males, because the mean recombination rate per chromosome is lower than in females.) Thus, only for this small subset of chromosomes can we assess the stringency of the rule assuring at least one chiasma for proper disjunction.

The robustness of our results for chromosome arms.

As shown in Supplementary Table 2, we have excellent marker coverage of each chromosomal arm, in both physical and genetic distance. Given the small proportion of crossover events that we could be missing, our estimates of the fraction of nullichiasmatic arms that segregate properly should be highly reliable.

As a further check, we reran our crossover-calling algorithm [4] requiring only two consecutive informative markers in order to call a crossover (i.e., K=2 rather than 5). Our previous analyses [4], notably the high concordance of our genetic map with that of Kong et al. (2002) [5] when setting K=5 [4], suggested that a threshold of K=2 likely leads to the inclusion of spurious crossover events that could incorrectly decrease our count of non-recombinant gametes. However, setting a less stringent threshold may decrease the false negative rate as well. In any case, the qualitative conclusion remained the same: nullichiasmatic chromosome arms frequently experience proper disjunction.

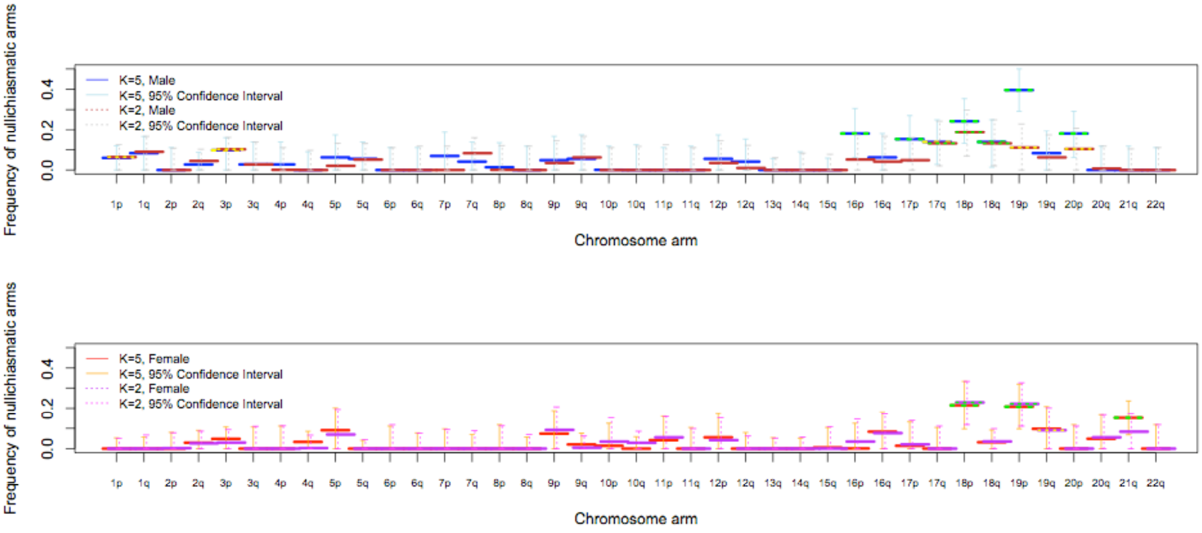

In addition, we examined whether incomplete marker coverage could be contributing to our findings by testing whether the mean number of crossovers identified in a family was associated with the distance from the centromere to the second or fifth to last informative marker (given that we used K=2 and 5 to call crossover events). Without correcting for multiple tests, results significant at the 5% level (by a Spearman rank correlation test) were 4p (p=0.004) and 19p (p=0.029) for the fifth to last informative in males. In females, they were 22q (p=0.027) for the fifth to last informative marker. We also tested for an association between the fraction of nullichiasmatic chromosomes in a family and the distance from the centromere to the second or fifth to last informative marker. Without correction for multiple tests, significant results at the 5% level (by a Spearman rank correlation test) for the fifth to last informative marker were 2p (p=0.033) and 10p (p=0.041) in males and 7q (p=0.005), 8q (p=0.024) and 16q (p=0.023) in females. Thus, there is little or no correspondence between the low p-values and chromosomes for which we found significant fractions of nullichiasmatic transmissions.

Robustness of our results for chromosomes.
The estimated fractions of nullichiasmatic chromosomes that experience proper disjunction are small and hence may be more sensitive to missing relatively few crossovers. Given the high density of markers in our data, we are highly unlikely to be missing double crossovers that might appear as non-recombinant gametes. However, we could be missing recombination events in the telomeres, past our last informative marker. Below we review the relevant properties of our data sets for the three chromosomes with significant evidence for nullichiasmatic transmissions when we assume complete marker coverage: chr. 12 and 18 in males and chr. 21 in females.

Chromosome 12 in males.
Across families, the median distance between the second informative marker on the p arm and the second to last informative marker on the q arm is 99.6% of the genetic length of the chromosome (based on Kong et al. (2002) [5]) and 99.6% of its physical

length, with all families lacking informative markers for 0.2-1.8cM. Given that we estimated that >0.1% of transmissions involved a nullichiasmatic chromosomes (our lowest 5%-tile), even the modest gap in our marker coverage renders the results highly tentative.

Chromosome 18 in males.
Across families, the median distance between the second informative marker on the p arm and the second to last informative marker on the q arm is 98.6% of the genetic length of the chromosome (based on Kong et al. (2002) [5]) and 99.4% of its physical length, with families lacking informative coverage for 0.9-2.6cM. If we assumed that all families lack marker coverage for 1.38 cM of the chromosome and that, conservatively, *all* the crossovers occurring in the missing region led to apparent non-recombinant gametes (i.e., that 0.0138x288 meioses should be moved from the 0 count bin to the 1 count bin), we still found a model without the transmission of nullichiasmatic chromosomes to be unlikely (p=0.036, based on 5000 permutations). However, given that there is incomplete marker coverage for the telomeric regions, where males experience high levels of recombination [2], the evidence for successful transmission of nullichiasmatic chromosomes 18 remains somewhat tentative.

Chromosome 21 in females.
We used two sets of data for female transmissions of chromosome 21: 288 meioses from a Hutterite pedigree data, that we had previously analyzed in Coop et al. (2008) [4] and 152 meioses from CEPH (European-American) pedigrees in which crossovers were called by Oliver et al. (2008) [6]. The two data sets differ in their marker coverage, with 6,624 single nucleotide polymorphisms (SNPs) genotyped in the Hutterite and 133 SNPs in the CEPH data, and we therefore discuss them separately.

Chromosome 21 is acrocentric, and all but ~280 kb of the short (p) arm is heterochromatic. Our data contain only four informative markers in the 280 kb, in part because the region is highly repetitive. We do not call any crossovers in it, whether K = 2 or 5. (In turn, the Oliver et al. data contain no markers on the p-arm.) The UCSC genome browser (http://genome.ucsc.edu/) provides a recombination rate of ~0.3 cM/Mb for this region (based on Ref. [5]), but the microsatellite markers on which that estimate relies appear to map to the long (q) arm (results not shown). Finally, a cytogenetic study of human female oocytes reported no chiasmata on the short arm of chromosome 21 in 60 bivalents with a single MLH1 focus, and one case of the 23 bivalents with two or more MLH1 foci, but even then the position of the centromere was uncertain [3]. Taken together, these lines of evidence indicate that we are highly unlikely to be over-estimating the number of non-recombinant gametes because of lack of coverage of the p arm.

Based on the Oliver et al. [6] data alone, the maximum likelihood estimate for 21q is 19.8% (lower 5%-tile: 6.7%, p= 0.0070). The span of all 133 markers does not include 4.4% of the genetic distance of 21q (based on Ref. [5]) and 5.6% of the physical distance. We estimate > 6.7% of transmissions involved a nullichiasmatic chromosome, which is still higher than 4.4% of the genetic distance missing on this chromosome.

Using the Hutterite data alone yields 12.5% as our estimate of the fraction of nullichiasmatic bivalents that segregated properly (lower 5%-tile: 2.8%, p= 0.0180). The median distance across families between the centromere and the second to last informative marker on the q arm is 100% of the genetic length of the chromosome (based on Kong et al. (2002) [5]), with families lacking informative markers for 0-2.1cM (except for one family with 4 offspring that lacks markers for 10cM). The median marker span in physical distance, in turn, is 97.8% of the arm. This represents excellent coverage, especially given the relatively low rate of female crossing-over in telomeric regions [2,3].

As an additional check on the Hutterite data, we reran our crossover-calling algorithm on chromosome 21 with K=2 instead of K=5 and examined each additional crossover that was inferred. Crossover events that are flanked on both sides by five informative markers in the family, but that are only supported by two of the five, are likely to be spurious [4]; we therefore discarded them. However, we retained all cases in which there were fewer than five informative markers between the putative crossover and the telomere (or the centromere), since we could not exclude the possibility that those are real events that we missed because of insufficient marker density towards the telomere (or centromere) when setting K=5. Including the additional events picked up for K=2, there are 135 cases of a non-recombinant chromosome 21 gamete, 126 cases of a gamete with one crossover and 27 gametes with two or more (instead of 138, 123 and 27 respectively, for K=5); the probability of the data under the null model with no nullichiasmatic transmissions becomes 0.046 (based on 5000 permutations).

Finally, to evaluate the robustness of our results for the two data sets jointly, we considered that all Oliver et al. families lack marker coverage for 4.4cM, and made the following, highly conservative assumptions: (i) That *all* Hutterite families are missing 2.1cM in coverage (when the median is actually 0 cM) and that four offspring are missing 10cM (iii) That *all* crossovers occurring in the missing regions led to apparent non-recombinant gametes. We then moved a total of 13 meioses (i.e., 0.044x152 + 0.021x284 + 0.4) from the 0 crossover bin to the 1 crossover bin. Having done so, we still found a model without the transmission of nullichiasmatic chromosomes to be unlikely (p=0.026, based on 5000 permutations). Thus, incomplete marker coverage cannot explain our findings for chromosome 21.

Characterizing crossover interference.

Following Broman and Weber (2000) [1], we estimated the parameter ν from our data, for each sex and each chromosome separately (Supplementary Figure 3). The value of ν estimated from the Hutterite data is similar to that obtained by Ref. [1], but with tighter confidence intervals due to the larger number of meioses available in the Hutterites.

Variation between chromosomes in the level of interference.

A likelihood ratio test (LRT) statistic comparing a model where the ν interference parameter is the same for all chromosomes to a model where each chromosome has a separate ν parameter. The LRT statistic = 2 ln(Likehood Ratio). The likelihoods for

the LRT test were calculated for the Hutterite genetic maps [4]. The 5% significance cutoff for a sex-specific LRT is 32.7.

	Males	Females
Hutterite map	166.5	124.1

Variation between the sexes in the level of interference.
A likelihood ratio test (LRT) statistic comparing a model where the sexes share the same ν parameter for a chromosome compared to a model where each sex has a separate ν parameter. The LRT statistic = 2 ln(Likehood Ratio). The 5% significance cutoff for chromosome specific LRT is 9.3, after Bonferroni correction for 22 tests. The likelihoods for the LRT test were calculated using the Kong et al. (2002) [5] and Hutterite genetic maps. Chromosomes for which there is a significant LRT at the 5% level are marked with an *.

Chr	Hutterite map
1	0.2
2 *	42
3	6.0
4	1.8
5	0.0
6	2.1
7	0.6
8	0.0
9	2.0
10	0.0
11 *	11.7
12	1.3
13 *	14.4
14	0.0
15	3.3
16	7.5
17	0.7
18	1.0
19 *	21.2
20	0.1
21 *	32.0
22	0.6
Total	148.9

As seen in these two tables, we find some evidence for variation between chromosomes and sexes in the strength of interference (i.e., variation in ν). However, given the lack of fit of the gamma model (main text Figure 4), these findings should be interpreted with caution.

Vetting double crossovers in close proximity.

To create a list of double crossovers in close proximity, we searched across transmissions for two events that occurred within 5 cM. The inter-crossover distances were calculated by linearly interpolating the Kong et al. (2002) [5] genetic map distances between the midpoints of the crossover intervals. A concern might be that this linear interpolation procedure introduces substantial error. To evaluate this possibility, we recalculated the distances between events using a genetic map estimated from linkage disequilibrium (LD) data instead of pedigree data [7]. The LD map is based on all meioses in the history of the sample, so has much greater spatial resolution; on the other hand, it is sex-averaged, making the direct comparison a bit difficult. Nonetheless, results are largely unchanged (not shown). Even using the Kong et al. (2002) distances between markers on either side of our crossover intervals (i.e., using no interpolation, at the cost of systematically over-estimating the true distance) yields an excess of close double crossovers (results not shown).

We also created a more stringently vetted list of double crossovers (see Supplementary Figure 4) by focusing only on events supported by all offspring in the nuclear family and by at least 15 or more high quality markers (i.e., excluding any marker in the bottom 5%-tile of the QC scores provided by the BLRMM genotype calling algorithm); see Supplementary Materials in Coop et al. (2008) [4] for details. Where available, we checked the grandparental genotypes to make sure that they were consistent with our haplotype reconstruction in the parents. We note that requiring 15 or more markers per crossover limits how closely spaced double crossovers can be in our data and in that respect, leads to an under-estimate of their proximity. In addition, we verified that there were no known segmental duplications and inversions in these regions (in the Segmental Duplications DB http://humanparalogy.gs.washington.edu/ and Database of Genomic Variants http://projects.tcag.ca/variation/). In 11 individuals, we observed a triple crossover within 5 cM, a pattern consistent with a crossover in an inversion homozygote [8]; five of these coincided with the location of a known polymorphic inversion [8,9]. The 11 cases were excluded from our stringent list. To assess the possibility of de novo deletions, in all cases where the double crossovers was within 5 Mb, we verified that the raw genotype intensities (kindly provided by D. Conrad) did not show a marked decrease in the region. No such cases were found. Finally, we excluded two cases of double crossovers that spanned the centromere.

A two pathway model for crossover interference

To further investigate the double crossovers in surprisingly close proximity, we implemented the two-pathway model of [10]. Under this model, a fraction (p) of recombination events is not subject to crossover interference, while the rest experiences interference according to the gamma model with strength parameter ν. In the tables below, we present estimates of the male and female parameter (p and ν) obtained for each chromosome, the estimate ν_0 where p is constrained to be zero, and the log-likelihood ratio comparing the model with p allowed to vary (p>0) to the null model with p=0. To evaluate the significance of the likelihood ratio for each chromosome, we conducted 1000 simulations under the null model p=0, given the genetic map length and ν_0 estimate for each chromosome. From these simulations, we

constructed a p-value from the number of simulations with a likelihood ratio greater than that observed for the chromosome (see [10]).

Estimates under two pathway model of interference and significance test.

Males

Chr.	ν	p	ν_0	ln LR testing p=0	p-value
1	9.96	0.10	3.84	15.08	0
2	12.46	0.13	2.04	44.87	0
3	6.79	0.03	5.05	2.78	0.006
4	9.39	0.05	5.15	8.11	0
5	7.16	0.03	4.90	4.53	0
6	10.80	0.06	4.46	14.77	0
7	9.83	0.06	4.44	14.98	0
8	13.33	0.14	2.21	30.90	0
9	10.50	0.12	2.96	13.57	0
10	9.37	0.06	4.73	7.34	0
11	10.82	0.03	7.15	3.99	0.003
12	9.35	0.05	3.54	20.85	0
13	11.65	0.10	3.48	13.52	0
14	6.75	0.03	4.17	6.83	0
15	9.42	0.02	6.62	4.19	0
16	6.83	0.03	4.91	1.51	0.019
17	4.76	0.06	3.31	1.85	0.013
18	4.53	0.00	4.53	0.00	1
19	4.55	0.08	2.88	1.50	0.024
20	6.47	0.07	3.88	2.44	0.007
21	Infinity	0.03	289.98	7.03	0.011
22	16.85	0.06	4.45	8.28	0.001
Total	9.17	0.08	3.70	260.48	

Females

Chr.	ν	p	ν_0	ln LR testing p=0	p-value
1	5.91	0.06	3.53	15.02	0
2	6.98	0.04	4.69	5.83	0.001
3	6.04	0.05	3.50	15.25	0
4	5.77	0.04	4.16	5.68	0
5	5.91	0.02	5.04	1.96	0.01
6	6.63	0.07	3.52	12.33	0
7	7.92	0.07	3.93	13.08	0
8	7.79	0.18	2.21	16.68	0
9	9.41	0.10	3.73	10.66	0
10	9.32	0.06	4.72	13.94	0
11	5.01	0.03	3.69	5.61	0.001
12	8.65	0.07	4.29	7.09	0
13	9.26	0.03	7.34	0.94	0.026
14	7.58	0.03	4.24	15.57	0

15	6.83	0.03	4.52	7.26	0.001
16	6.96	0.12	2.81	10.27	0
17	7.74	0.07	3.88	9.56	0
18	6.95	0.02	5.61	0.83	0.032
19	8.42	0.00	8.42	0.00	1
20	7.30	0.10	3.66	3.26	0.004
21	9.89	0.09	2.82	5.38	0
22	9.53	0.10	3.59	2.04	0.011
Total	6.96	0.06	3.83	198.50	

Modeling the effects of chromatid interference.

In our analysis, we assumed that when multiple chiasmata occur on a bivalent, the pairs of chromatids involved in each chiasma are chosen at random and independently. Departures from this assumption are referred to as chromatid interference. In the most extreme case of positive chromatid interference, all chiasmata could form between the same pair of chromatids, leading the number of crossovers, Y, in a randomly chosen gamete to equal the number of chiasmata in the tetrad, X, or zero, with equal probability. More generally, chromatid interference can increase the variance in the number of crossovers in a gamete (Y) given the number of chiasmata in the tetrad (X) relative to the no interference model.

A simple, non-mechanistic model that exhibits this basic feature of chromatid interference is provided by the beta-binomial distribution, which can describe the number of crossovers, Y, in a gamete given X chiasmata in the bivalent:

$$\Pr(Y = y \mid X = x) = \binom{x}{y} \frac{\mathrm{B}(y + 1/k, x - y + 1/k)}{\mathrm{B}(1/k, 1/k)}, \quad \text{(S1)}$$

where B() is the beta function. (*Cf.* Equation 1 of the main text.) The parameter k controls the variance in the number of crossovers given the number of chiasmata and can thus be viewed as controlling the degree of chromatid interference; $k = 0$ corresponds to the case of no interference. For the results obtained using this approach, see Supplementary Figure 5.

To infer the parameter k in the maximum likelihood setting, we adapted the EM algorithm of Ott (1996) [11] as follows. Writing Y_i for the observed number of crossovers in gamete i ($i = 1\ldots N$), and X_i for the unobserved number of chiasmata in the underlying tetrad, the log-likelihood for the *complete* data (observed and unobserved) is

$$\begin{aligned} l(p,k) = \sum_{i=1}^{N} \log p_{X_i} + \log\binom{X_i}{Y_i} \\ + \log \mathrm{B}(Y_i + 1/k, X_i - Y_i + 1/k) - \log \mathrm{B}(1/k, 1/k). \end{aligned} \quad \text{(S2)}$$

Therefore the E step of the EM algorithm [12] can be written

$$E\left[l(p,k)\,|\,Y,p^{(m)},k^{(m)}\right] = \sum_{x=0}^{x_{\max}}\sum_{y=0}^{x} E_{xy}\left[\log p_x + \log\binom{x}{y} + \log \mathrm{B}\left(y+1/k, x-y+1/k\right) - \log \mathrm{B}\left(1/k, 1/k\right)\right], \tag{S3}$$

where $p^{(m)}$ and $k^{(m)}$ are the parameter values from the previous iteration and

$$\begin{aligned} E_{xy} &= n_y \Pr\left(X_i = x\,|\,Y_i = y, p^{(m)}, k^{(m)}\right) \\ &= \frac{n_y p_x^{(m)} \binom{x}{y} \mathrm{B}\left(y+1/k^{(m)}, x-y+1/k^{(m)}\right)}{\sum_{x'=y}^{x_{\max}} p_{x'}^{(m)} \binom{x'}{y} \mathrm{B}\left(y+1/k^{(m)}, x'-y+1/k^{(m)}\right)}, \end{aligned} \tag{S4}$$

where n_y is the number of gametes with y crossovers observed. Then in the M-step,

$$p_x^{(m+1)} = \frac{1}{N}\sum_{y=0}^{x} E_{xy}$$

and (S5)

$$k^{(m+1)} = \arg\max_k \sum_{x=0}^{x_{\max}}\sum_{y=0}^{x} E_{xy}\left[\log \mathrm{B}\left(y+1/k, x-y+1/k\right) - \log \mathrm{B}\left(1/k, 1/k\right)\right]$$

is solved numerically. Starting from arbitrary p and k, the algorithm is iterated using Equation S5 until convergence to the desired precision.

Testing for transmission distortion of genotypes on chromosome 21.

To examine the possibility that certain genotypes are preferentially transmitted on chromosome 21 in females, we performed transmission distortion tests (tdt) to assess the deviation from Mendelian segregation in the transmissions from heterozygote mothers, using the Hutterite data. To this end, we used the parent of origin flag "--tdt --poo" in PLINK [13] to calculate the asymptotic p-values for the tdt test, treating all children as affected.

Quantile plot of the p-values from a test of maternal transmission distortion for all SNPs on chromosome 21.

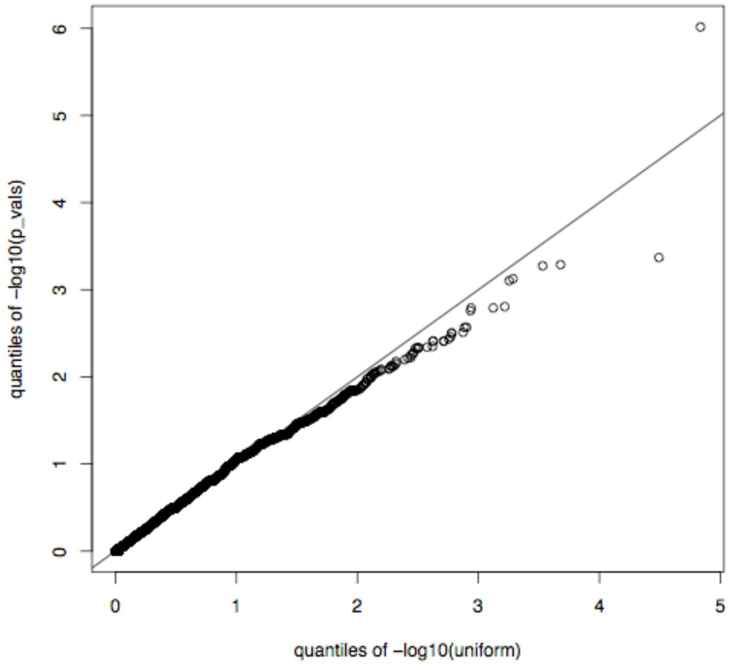

(The point in the top right is a SNP that had an exceptionally high rate of genotyping error.)

As can be seen, the p-values closely follow a uniform distribution, suggesting that there is no strong evidence of transmission distortion. Moreover, there is no enrichment of significant p-values close to the telomere or centromere of chromosome 21 (regions of the genome that are though to be particularly susceptible to meiotic drive); for example, ~5% of the 100 first and last markers on the chromosome have a p-value <0.05.

A Bayesian approach to assess the support for proper disjunction of nullichiasmatic bivalents.
We devised an MCMC scheme to explore the posterior distribution of parameters and models in a Bayesian setting; for the results, see Supplementary Figure 1.

To that end, we considered the following models:

Model m = 0	No chromatid interference (k = 0)<br>Nullichiasmatic bivalents are allowed ($p_0 \geq 0$)
Model m = 1	No chromatid interference (k = 0)<br>Nullichiasmatic bivalents are assumed inviable (p_0 = 0)
Model m = 2	Chromatid interference ($k \geq 0$)<br>Nullichiasmatic bivalents are assumed inviable (p_0 = 0)

We did not fit a model that allowed for both nullichiasmatic bivalents and chromatid interference because we considered these as competing explanations for an excess of gametes with zero crossovers.

The object of inference was the posterior distribution of model (m) and parameters (p, k), which can be factorized as follows:

$$f\left(p,k,m \mid Y\right) \propto \Pr\left(Y \mid p,k,m\right) f\left(p,k \mid m\right) \Pr\left(m\right), \qquad \text{(S6)}$$

where $\Pr\left(Y \mid p,k,m\right)$ is the likelihood of the observed data Y

$$\Pr\left(Y \mid p,k,m\right) = \begin{cases} \displaystyle\prod_{i=1}^{N} \sum_{x=Y_i}^{x_{\max}} p_x \binom{x}{Y_i} \frac{1}{2^x} & m = 0 \text{ or } 1 \\ \displaystyle\prod_{i=1}^{N} \sum_{x=Y_i}^{x_{\max}} p_x \binom{x}{Y_i} \frac{\mathrm{B}\left(Y_i + 1/k, x - Y_i + 1/k\right)}{\mathrm{B}\left(1/k, 1/k\right)} & m = 2 \end{cases} \qquad \text{(S7)}$$

$f\left(p,k \mid m\right)$ is the prior distribution, which is

$$f\left(p,k \mid m\right) = \begin{cases} \dfrac{\mu \mathrm{e}^{-\mu k}}{\mathrm{B}\left(\alpha_{0\ldots x_{\max}}\right)} \displaystyle\prod_{x=0}^{x_{\max}} p_x^{\alpha_x - 1} & m = 0 \\ \dfrac{\mu \mathrm{e}^{-\mu k}}{\mathrm{B}\left(\alpha_{1\ldots x_{\max}}\right)} \displaystyle\prod_{x=1}^{x_{\max}} p_x^{\alpha_x - 1} & m = 1 \text{ or } 2 \end{cases} \qquad \text{(S8)}$$

when assuming an exponential prior with rate $\mu = 10$ for the chromatid interference parameter, and $\Pr\left(m\right)$ is the prior model probability (which we set uniform across models).

When applying MCMC to proportion parameters such as p, which is constrained to sum to one, the chain is susceptible to numerical rounding errors that can cause failure of the algorithm. To overcome this problem, we used an equivalent reparameterization of p based on the following relationship between the Dirichlet distribution and the gamma distribution: if φ_x, $x = 0\ldots x_{\max}$ are independent gamma random variables with shape parameters α_x and scale parameter 1, and

$$p_x = \varphi_x \Big/ \sum\nolimits_{x'=0}^{x_{\max}} \varphi_{x'}, \qquad \text{(S9)}$$

then the vector p has the Dirichlet distribution with parameter vector α. Therefore we assumed that, *a priori*, the φ_x's follows an independent gamma distribution with parameters (α_x, 1). For Models 1 and 2, we imposed the constraint that $p_0 = 0$ regardless of the value of φ_0, which allows φ_0 to follow its prior gamma distribution when $m = 1$ or 2.

$$f\left(\varphi,k \mid m\right) = \mu \mathrm{e}^{-\mu k} \prod_{x=0}^{x_{\max}} \frac{\varphi_x^{\alpha_x - 1} \mathrm{e}^{-\varphi_x}}{\Gamma\left(\alpha_x\right)} \qquad \text{(S10)}$$

where $\Gamma(\alpha)$ is the gamma function. The likelihood is calculated by performing the transformation given by Equation S19.

Three types of Metropolis-Hastings move were implemented: a Log-normal proposal in which one of the φ_x's, or k, was perturbed by a random amount, a switching proposal in which the values were switched between a pair of the φ_x's, and a model switching move.

Log-normal move. For example, an update to φ_x' from φ_x is proposed: Let $\varphi_x' \sim \text{LogNormal}(\varphi_x, \sigma_\varphi)$. This move is accepted with probability

$$\alpha(\varphi_x \to \varphi_x') = \min\left\{1, \frac{\Pr(Y \mid p', k, m)}{\Pr(Y \mid p, k, m)} \frac{f(\varphi_x', k \mid m)}{f(\varphi_x, k \mid m)} \frac{\varphi_x'}{\varphi_x}\right\}. \quad \text{(S11)}$$

Here σ_φ is a parameter that controls the variance of the proposal move, and can be tuned to improve mixing of the MCMC. An analogous move is also used to update k.

Switching move. For example, it is proposed to switch φ_x with φ_y. Let $\varphi_x' = \varphi_y$ and $\varphi_y' = \varphi_x$. This move is accepted with probability

$$\alpha(\varphi \to \varphi') = \min\left\{1, \frac{\Pr(Y \mid p', k, m)}{\Pr(Y \mid p, k, m)} \frac{f(\varphi', k \mid m)}{f(\varphi, k \mid m)}\right\}. \quad \text{(S12)}$$

Model switching move. The model is switched from m to m' where m' is chosen uniformly at random from the models other than m. The acceptance probability is

$$\alpha(m \to m') = \min\left\{1, \frac{\Pr(Y \mid p, k, m')}{\Pr(Y \mid p, k, m)} \frac{f(\varphi, k \mid m')}{f(\varphi, k \mid m)} \frac{\Pr(m')}{\Pr(m)}\right\}. \quad \text{(S13)}$$

In practice, when the MCMC moves to a model with fewer parameters, for example from Model 2 to 1, in which case parameter k is lost, updates to k are still proposed and k follows its prior distribution. Under Models 0 and 1, k has no effect on the likelihood and therefore the correct posterior distributions are obtained.

Model comparison using Bayes factors

To compare the relative likelihood of each pair of models, which is equivalent to the posterior odds since $\Pr(m) = 1/3$, we calculated Bayes factors by averaging over the parameters p and (where appropriate) k. For example, the Bayes factor for Model 2 versus Model 1 was calculated as the ratio of marginal likelihoods,

$$B_{1,2} = \frac{P(Y \mid m = 2)}{P(Y \mid m = 1)} = \frac{\iint \Pr(Y \mid p, k, m = 2) f(p, k \mid m = 2) \mathrm{d}p \mathrm{d}k}{\iint \Pr(Y \mid p, k, m = 1) f(p, k \mid m = 1) \mathrm{d}p \mathrm{d}k}. \quad \text{(S14)}$$

In practice, this quantity was computed simply as the relative number of iterations of the MCMC for which $m = 2$ *versus* $m = 1$. When $B_{1,2} > 1$ that suggests the data are better supported by Model 2 than Model 1, and *vice versa* when $B_{1,2} < 1$. One benefit of using Bayes factors rather than maximum likelihood ratios is that non-nested models

can be compared. Bayes factors are, however, sensitive to the choice of prior, and for this reason we analyzed the sensitivity of the Bayes factors to our choice.

A maternal age effect on recombination patterns? Maternal age is known to be an important risk factor for non-disjunction and accordingly, the recombination patterns underlying trisomy change with maternal age [6]. We were interested in examining whether recombination patterns underlying proper disjunction might also show an age effect. We therefore focused on transmissions in mothers older than 35 at birth vs. younger than 35 (maternal ages at birth were kindly provided to us for the Oliver et al. data [6] by Tiffany R. Oliver). As can be seen below, the estimated fraction of nullichiasmatic chromosomes does not differ significantly between groups.

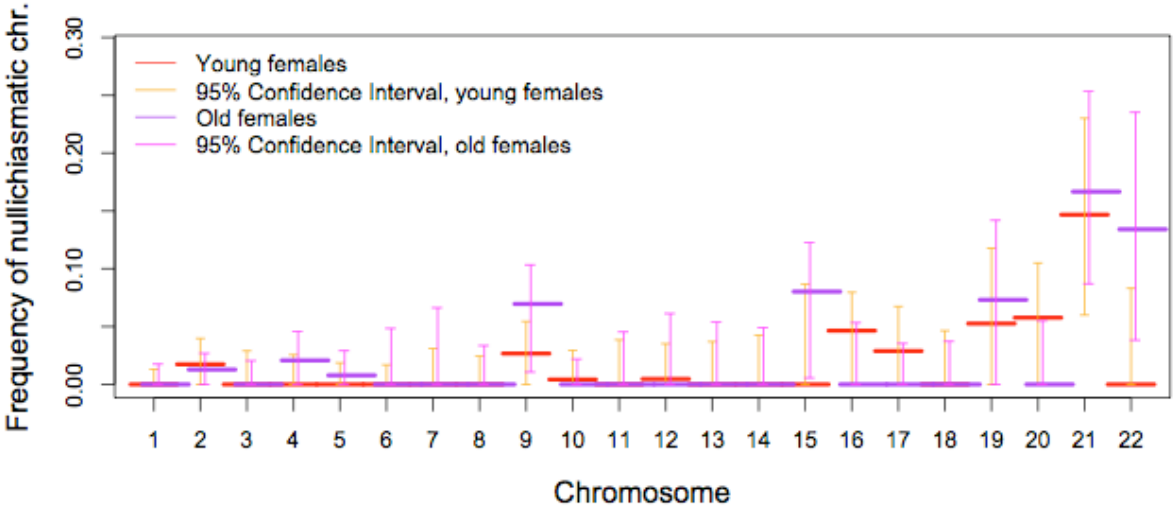

Similarly, the median distance from the centromere to the first crossover did not differ significantly between the two groups (correcting for multiple tests, all p-values > 0.05; results not shown). However, this could simply reflect lack of power, given that we only have data about 41 transmissions in older mothers (72 for chromosome 21).

References

1. Broman KW, Weber JL (2000) Characterization of human crossover interference. Am J Hum Genet 66: 1911-1926.
2. Lynn A, Ashley T, Hassold T (2004) Variation in human meiotic recombination. Annu Rev Genomics Hum Genet 5: 317-349.
3. Tease C, Hartshorne GM, Hulten MA (2002) Patterns of meiotic recombination in human fetal oocytes. Am J Hum Genet 70: 1469-1479.
4. Coop G, Wen W, Ober C, Pritchard J, Przeworski M (2008) High resolution mapping of crossovers reveals extensive variation in fine-scale recombination patterns among humans. Science 319: 1395-1398.
5. Kong A, Gudbjartsson DF, Sainz J, Jonsdottir GM, Gudjonsson SA, et al. (2002) A high-resolution recombination map of the human genome. Nat Genet 31: 241-247.
6. Oliver TR, Feingold E, Yu K, Cheung V, Tinker S, et al. (2008) New insights into human nondisjunction of chromosome 21 in oocytes. PLoS Genet 4: e1000033.
7. McVean GA, Myers SR, Hunt S, Deloukas P, Bentley DR, et al. (2004) The fine-scale structure of recombination rate variation in the human genome. Science 304: 581-584.
8. Broman KW, Matsumoto N, Giglio S, Martin CL, Roseberry JA, et al. (2003) Common long human inversion polymorphism on chromosome 8p. In: Goldstein DR, editor. Science and Statistics: A Festschrift for Terry Speed pp. 237-245.
9. Kidd JM, Cooper GM, Donahue WF, Hayden HS, Sampas N, et al. (2008) Mapping and sequencing of structural variation from eight human genomes. Nature 453: 56-64.
10. Housworth EA, Stahl FW (2003) Crossover interference in humans. Am J Hum Genet 73: 188-197.
11. Ott J (1996) Genetic mapping and DNA sequencing. In: Speed T, Waterman MS, editors. The IMA Volumes in Mathematics and its Applications. New York: Springer Verlag. pp. 49-63.
12. Dempster A, Laird N, Rubin D (1977) Maximum likelihood from incomplete data via the EM algorithm. Journal of the Royal Statistical Society Series B 39: 1-38.
13. Purcell S, Neale B, Todd-Brown K, Thomas L, Ferreira MA, et al. (2007) PLINK: a tool set for whole-genome association and population-based linkage analyses. Am J Hum Genet 81: 559-575.
14. Mancera E, Bourgon R, Brozzi A, Huber W, Steinmetz LM (2008) High-resolution mapping of meiotic crossovers and non-crossovers in yeast. Nature 454: 479-485.

Supplementary Table 1

	chr	X0	X1	X2	X3	X4	X5	X6	X7	X8	Span[+]
females	1	3	27	74	67	54	40	20	2	1	3
	2	13	32	70	68	62	30	9	3	1	1
	3	12	53	80	73	49	20	1	0	0	0
	4	15	55	83	74	44	10	7	0	0	3
	5	13	60	93	75	37	8	2	0	0	4
	6	12	64	81	86	36	7	2	0	0	0
	7	18	79	78	78	28	5	2	0	0	2
	8	15	85	97	56	24	7	4	0	0	1
	9	31	70	99	68	12	8	0	0	0	12
	10	18	67	99	77	21	5	1	0	0	1
	11	27	97	92	57	11	3	1	0	0	1
	12	26	76	99	59	22	6	0	0	0	2
	13	37	122	97	30	2	0	0	0	0	0
	14	43	114	108	21	2	0	0	0	0	0
	15	57	108	82	38	3	0	0	0	0	0
	16	44	92	102	43	7	0	0	0	0	1
	17	42	91	123	27	4	1	0	0	0	2
	18	44	116	91	31	5	1	0	0	0	2
	19	80	126	72	10	0	0	0	0	0	4
	20	58	117	90	22	1	0	0	0	0	0
	21	138	123	24	3	0	0	0	0	0	0
**	21	79	60	12	1	0	0	0	0	0	0
	22	117	144	23	4	0	0	0	0	0	0
males	1	23	78	95	64	27	1	0	0	0	1
	2	20	83	103	61	16	5	0	0	0	1
	3	31	86	117	45	8	1	0	0	0	0
	4	33	119	103	32	1	0	0	0	0	0
	5	44	116	88	38	2	0	0	0	0	1
	6	41	123	94	29	1	0	0	0	0	0
	7	44	114	96	32	2	0	0	0	0	0
	8	56	127	82	19	4	0	0	0	0	0
	9	52	138	73	23	2	0	0	0	0	0
	10	47	134	83	23	1	0	0	0	0	0
	11	51	136	86	15	0	0	0	0	0	0
	12	60	96	103	28	1	0	0	0	0	0
	13	53	147	82	5	1	0	0	0	0	0
	14	76	145	64	3	0	0	0	0	0	0
	15	69	143	73	3	0	0	0	0	0	0
	16	87	131	65	5	0	0	0	0	0	1
	17	80	140	64	4	0	0	0	0	0	0
	18	101	123	62	2	0	0	0	0	0	0
	19	100	143	41	4	0	0	0	0	0	0
	20	80	147	59	2	0	0	0	0	0	0
	21	134	154	0	0	0	0	0	0	0	0
	22	121	150	17	0	0	0	0	0	0	0

For each chromosome or chromosome arm, the observed number of transmissions with a given number of crossovers (where Xi denotes the number with *i* crossovers).
** Data from Oliver et al. (2008)[6]
+ Span refers to the number of events localized within intervals that span the centomere gap boundary. We conservatively assigned such events to both arms when testing for a non-zero fraction of nullichiasmatic transmissions.

	chr	arm	X0	X1	X2	X3	X4	X5
females	1	p	30	100	89	51	16	2
	2	p	57	121	83	25	2	0
	3	p	63	115	87	21	2	0
	4	p	104	153	26	5	0	0
	5	p	130	131	27	0	0	0
	6	p	82	144	53	8	1	0
	7	p	82	139	61	6	0	0
	8	p	112	139	26	7	4	0
	9	p	112	132	42	1	1	0
	10	p	113	142	33	0	0	0
	11	p	123	136	27	2	0	0
	12	p	134	136	18	0	0	0
	16	p	115	143	29	1	0	0
	17	p	138	142	8	0	0	0
	18	p	169	113	6	0	0	0
	19	p	168	114	6	0	0	0
	20	p	129	146	13	0	0	0
	1	q	38	116	92	38	3	1
	2	q	42	88	89	57	9	3
	3	q	59	118	78	32	1	0
	4	q	39	86	97	51	14	1
	5	q	30	98	102	46	10	2
	6	q	47	125	80	32	4	0
	7	q	63	131	75	16	3	0
	8	q	49	135	78	21	4	1
	9	q	61	124	85	17	1	0
	10	q	47	111	103	22	4	1
	11	q	71	132	66	18	1	0
	12	q	50	114	90	31	3	0
	13	q	37	122	97	30	2	0
	14	q	43	114	108	21	2	0
	15	q	57	108	82	38	3	0
	16	q	99	130	57	2	0	0
	17	q	81	138	62	7	0	0
	18	q	77	135	72	2	2	0

	19	q	134	130	24	0	0	0
	20	q	123	136	28	1	0	0
	21	q	138	123	24	3	0	0
**	21	q	79	60	12	1	0	0
	22	q	117	144	23	4	0	0
males	1	p	76	126	77	9	0	0
	2	p	102	141	38	6	1	0
	3	p	91	124	71	2	0	0
	4	p	147	140	1	0	0	0
	5	p	153	135	0	0	0	0
	6	p	127	154	7	0	0	0
	7	p	150	134	4	0	0	0
	8	p	142	140	3	3	0	0
	9	p	147	137	4	0	0	0
	10	p	141	144	3	0	0	0
	11	p	140	148	0	0	0	0
	12	p	150	136	2	0	0	0
	16	p	165	118	5	0	0	0
	17	p	162	122	4	0	0	0
	18	p	178	109	1	0	0	0
	19	p	198	87	3	0	0	0
	20	p	167	118	3	0	0	0
	1	q	92	129	64	3	0	0
	2	q	68	126	85	9	0	0
	3	q	110	138	38	2	0	0
	4	q	77	143	64	4	0	0
	5	q	85	131	67	5	0	0
	6	q	98	147	42	1	0	0
	7	q	89	134	61	4	0	0
	8	q	111	148	26	3	0	0
	9	q	114	134	38	2	0	0
	10	q	103	149	34	2	0	0
	11	q	111	150	26	1	0	0
	12	q	97	135	53	3	0	0
	13	q	53	147	82	5	1	0
	14	q	76	145	64	3	0	0
	15	q	69	143	73	3	0	0
	16	q	146	135	7	0	0	0
	17	q	151	124	13	0	0	0
	18	q	155	124	9	0	0	0
	19	q	150	132	6	0	0	0
	20	q	142	145	1	0	0	0
	21	q	134	154	0	0	0	0

22	q	121	150	17	0	0	0

For each chromosome or chromosome arm, the observed number of transmissions with a given number of crossovers (where Xi denotes *i* crossovers).
** Data from Oliver et al. (2008)[6]

Supplementary Table 2

	Chr	Arm	Median (bps)	Total (bps)	Median* in %	Median (cM)	Total (cM)	Median+ in %
Male								
	1	p	119650750	121236957	0.987	94.542	96.098	0.984
	1	q	121382370	122045891	0.995	101.908	101.978	0.999
	2	p	91489823	91689898	0.998	81.054	81.911	0.990
	2	q	147507080	148328332	0.994	109.412	109.412	1.000
	3	p	90507573	90587544	0.999	83.727	83.920	0.998
	3	q	105314899	106018197	0.993	79.872	80.543	0.992
	4	p	49247894	49354874	0.998	53.569	53.840	0.995
	4	q	138534845	139056345	0.996	95.889	96.025	0.999
	5	p	46103522.5	46441398	0.993	53.910	54.570	0.988
	5	q	131075643	131416469	0.997	95.623	95.623	1.000
	6	p	58770087	58938125	0.997	57.562	58.040	0.992
	6	q	108704895	109037575	0.997	77.580	77.580	1.000
	7	p	57869185	58058273	0.997	54.924	56.383	0.974
	7	q	97448543	97569867	0.999	79.150	79.150	1.000
	8	p	43728729	43958052	0.995	49.960	49.960	1.000
	8	q	98999801	99316775	0.997	68.988	69.032	0.999
	9	p	46914019	47107499	0.996	51.522	51.910	0.993
	9	q	87995118	88321770	0.996	69.388	69.388	1.000
	10	p	38900641	39244941	0.991	51.566	52.140	0.989
	10	q	93533896	93788688	0.997	81.750	81.750	1.000
	11	p	51228184	51450781	0.996	47.500	47.500	1.000
	11	q	79819138	80001604	0.998	61.860	61.860	1.000
	12	p	34621481	34747961	0.996	53.066	53.495	0.992
	12	q	95976952	96306851	0.997	82.127	82.127	1.000
	13	q	95477281	96274981	0.992	102.911	103.067	0.998
	14	q	86752683	88298586	0.982	94.626	95.451	0.991
	15	q	81187132	82078916	0.989	103.340	103.919	0.994
	16	p	35001914	35143302	0.996	52.992	53.630	0.988
	16	q	51611826	51883953	0.995	54.413	54.413	1.000
	17	p	22113712	22187133	0.997	47.781	48.212	0.991
	17	q	56215314	56487610	0.995	61.540	61.540	1.000
	18	p	15137683	15400898	0.983	41.957	43.055	0.974
	18	q	59162399	59352258	0.997	57.237	57.373	0.998
	19	p	26623880	26923622	0.989	41.014	41.100	0.998
	19	q	33667247	33888030	0.993	51.540	51.540	1.000
	20	p	26106047	26267569	0.994	48.479	50.266	0.964
	20	q	34306951	34402735	0.997	24.982	24.982	1.000
	21	q	32890384	33684324	0.976	47.521	47.521	1.000
	22	q	33865003	35224711	0.961	49.087	49.318	0.995
Female	1	p	119673538	121236957	0.987	184.104	186.037	0.990
	1	q	121371981	122045891	0.994	162.249	162.457	0.999

2	p	91548435	91689898	0.998	142.326	142.588	0.998
2	q	147554680	148328332	0.995	184.179	184.179	1.000
3	p	90507573	90587544	0.999	136.060	136.230	0.999
3	q	105448421	106018197	0.995	141.304	141.338	1.000
4	p	49026024	49354874	0.993	78.987	78.987	1.000
4	q	138624323	139056345	0.997	179.420	179.420	1.000
5	p	46199680	46441398	0.995	80.000	80.000	1.000
5	q	131043621	131416469	0.997	179.983	179.983	1.000
6	p	58769542	58938125	0.997	102.363	102.363	1.000
6	q	108691687	109037575	0.997	138.604	138.604	1.000
7	p	57897426	58058273	0.997	99.690	99.730	1.000
7	q	97401348.5	97569867	0.998	129.792	129.792	1.000
8	p	43730720	43958052	0.995	72.233	72.233	1.000
8	q	99077926	99316775	0.998	139.290	139.290	1.000
9	p	46843392	47107499	0.994	70.799	70.882	0.999
9	q	87887729	88321770	0.995	121.056	121.056	1.000
10	p	38916389	39244941	0.992	74.728	75.500	0.990
10	q	93454511	93788688	0.996	144.980	144.980	1.000
11	p	51228184	51450781	0.996	80.401	80.401	1.000
11	q	79916880	80001604	0.999	115.090	115.090	1.000
12	p	34642588.5	34747961	0.997	57.882	57.882	1.000
12	q	95991703	96306851	0.997	148.264	148.264	1.000
13	q	95469246	96274981	0.992	155.880	155.880	1.000
14	q	86711951	88298586	0.982	141.876	142.360	0.997
15	q	81030706	82078916	0.987	154.859	154.873	1.000
16	p	34962092	35143302	0.995	60.076	60.541	0.992
16	q	51674815.5	51883953	0.996	89.852	89.852	1.000
17	p	22075911	22187133	0.995	53.183	53.184	1.000
17	q	56209614	56487610	0.995	106.935	106.935	1.000
18	p	15184414	15400898	0.986	38.945	38.979	0.999
18	q	59270463	59352258	0.999	101.063	101.063	1.000
19	p	26605534	26923622	0.988	54.705	54.705	1.000
19	q	33667373	33888030	0.993	70.180	70.180	1.000
20	p	26140714	26267569	0.995	59.380	59.380	1.000
20	q	34216507	34402735	0.995	61.896	61.896	1.000
21	q	32946532	33684324	0.978	76.903	76.903	1.000
21^	q	31801920	33684324	0.866	73.193	76.537	0.956
22	q	33779283	35224711	0.959	82.920	83.217	0.996

For a chromosome, the span of marker coverage is measured as the distance between the second informative marker on the p arm to the second to last informative marker on the q arm (based on build May 2004 from the UCSC genome browser http://genome.ucsc.edu/). For a chromosome arm, the span of marker coverage is measured as the distance from the second to last informative marker to the endpoints of the centromere gap boundary, as defined in the UCSC genome browser.

* The median span across Hutterite families (weighted by offspring number), as a

percent of the total physical length.
+ The median span across Hutterite families (weighted by offspring number), as a percent of the total genetic length of the arm based on Kong et al. (2002)[5], the most precise genetic map available for humans.
^ Oliver et al. (2008)[6] data. For this study, we report the total span of all markers.

Supplementary Table 3

A list of stringently vetted double crossovers observed in the Hutterites. Shown are labels for the nuclear family and individual, the physical locations of double crossovers and the physical and genetic distance between them (i.e., using the midpoints of the two intervals in which the crossovers are localized). See the Supplementary Methods for details of the vetting procedure.

	Chr.	Family	Ind.	Start Interval (bp)	End Interval (bp)	Distance (cM)	Distance (Mb)
Males	1	A	1	12197058	12352966		
	1	A	1	15063705	15239216	2.70	2.88
	1	B	1	86712329	86740876		
	1	B	1	91838623	91931089	3.26	5.16
	1	C	1	9401069	9571857		
	1	C	1	10551300	10767127	1.46	3.40
	2	D	1	108240642	109381620		
	2	D	1	118734753	118745309	4.95	9.93
	2	E	1	52232228	52422338		
	2	E	1	56004263	56078481	1.94	3.71
	4	F	1	8233231	8305513		
	4	F	1	11872842	11899834	4.94	3.62
	6	W	1	12332702	12403489		
	6	W	1	13814229	13863564	1.09	1.47
	6	H	1	102581232	102813768		
	6	H	1	105864431	106081913	1.46	3.28
	7	I	1	146859724	146862110		
	7	I	1	149530332	149623439	3.05	2.72
	11	G	1	71861792	72673560		
	11	G	1	80689139	80889529	4.17	8.52
	12	J	1	48563396	48621353		
	12	J	1	50312811	50337397	0.76	1.73
	12	K	1	114016078	114074255		
	12	K	1	117409243	117479038	2.94	3.40
	19	H	2	62191356	62437884		
	19	H	2	63170308	63172291	0.00	0.86
Females	1	N	1	150149091	150662242		
	1	N	1	151557880	151880356	1.78	1.31
	1	O	1	43096311	43155717		
	1	O	1	43951297	44179221	1.15	0.94
	1	P	1	54453788	54474742		
	1	P	1	56511820	56531172	3.64	2.06
	2	L	2	36929203	36960938		
	2	L	2	39513540	39743590	4.61	2.68

2	Q	1	200426527	200446269		
2	Q	1	203380849	204188811	3.56	3.35
3	O	1	62086514	62088317		
3	O	1	64447283	64518944	4.48	2.40
6	R	1	20358892	21126634		
6	R	1	22143561	22263420	3.02	1.46
7	O	2	155176229	155241348		
7	O	2	158519631	158604512	1.90	3.35
8	W	2	6949821	8147895		
8	W	2	8985925	9200944	2.14	4.45
10	W	3	125440482	125693013		
10	W	3	126834513	127166639	4.83	1.43
11	X	1	98765668	99128324		
11	X	1	100255370	101008705	2.30	1.69
12	Y	1	129653804	129669889		
12	Y	1	132282738	132352440	1.51	2.66
14	Z	1	57611159	58278190		
14	Z	1	60217834	60771253	2.12	2.55
16	W	1	79738937	79799603		
16	W	1	80474139	80474600	2.73	0.71

Supplementary Figure 1

A) For each chromosomal arm, the estimated fraction of bivalents that segregated properly without a chiasma, in male (blue) and female (red) transmissions. Shown are the modes of the estimated posterior distributions and the 95% credible intervals. The data for chromosome 21 combine two sets of pedigrees (see main text Methods). Results are for a prior distribution with J=1 (see main text Methods).

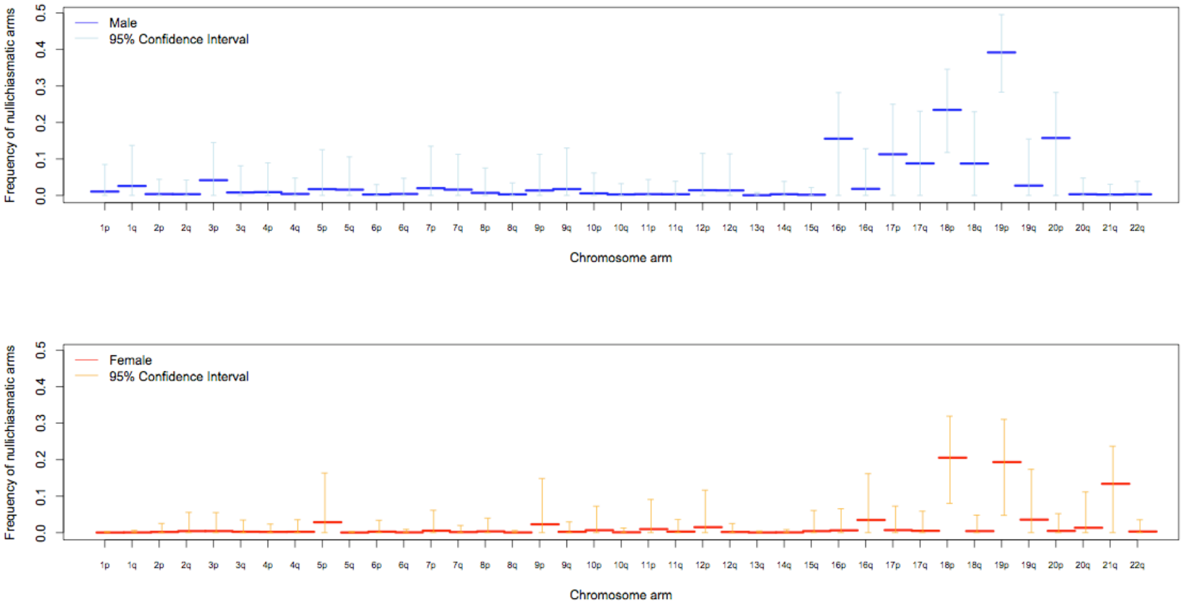

B) For each chromosomal arm, the Bayes Factor in support of a model with nullichiasmatic chromosomes vs. a model with an obligate crossover, for males (blue) and females (red). See Supplementary Methods for details.

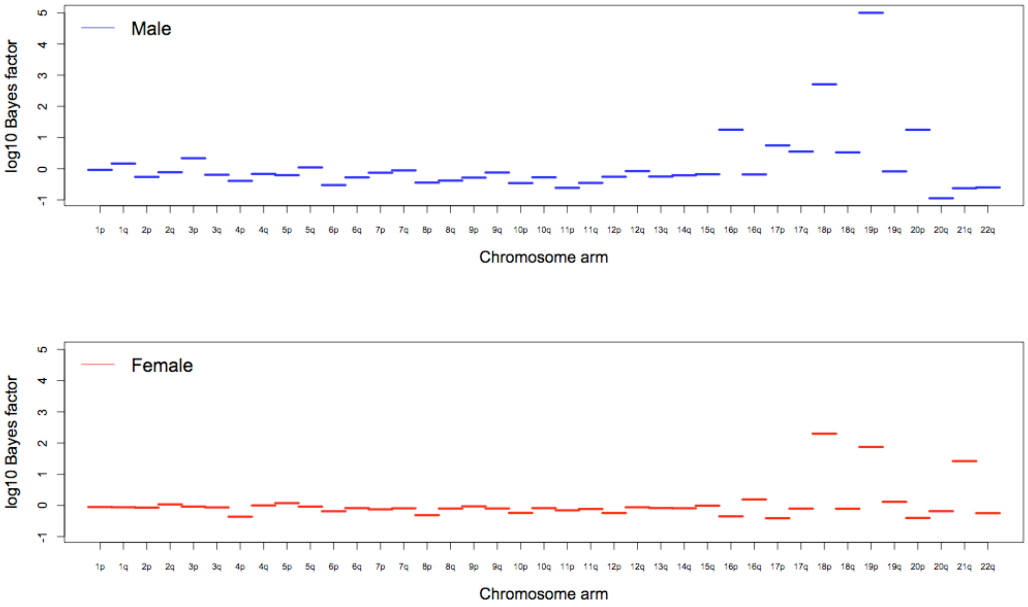

C) For each chromosome, the estimated fraction of bivalents that segregated properly without a chiasma, in male (blue) and female (red) transmissions. See the legend of Supplementary Figure 1A for additional details.

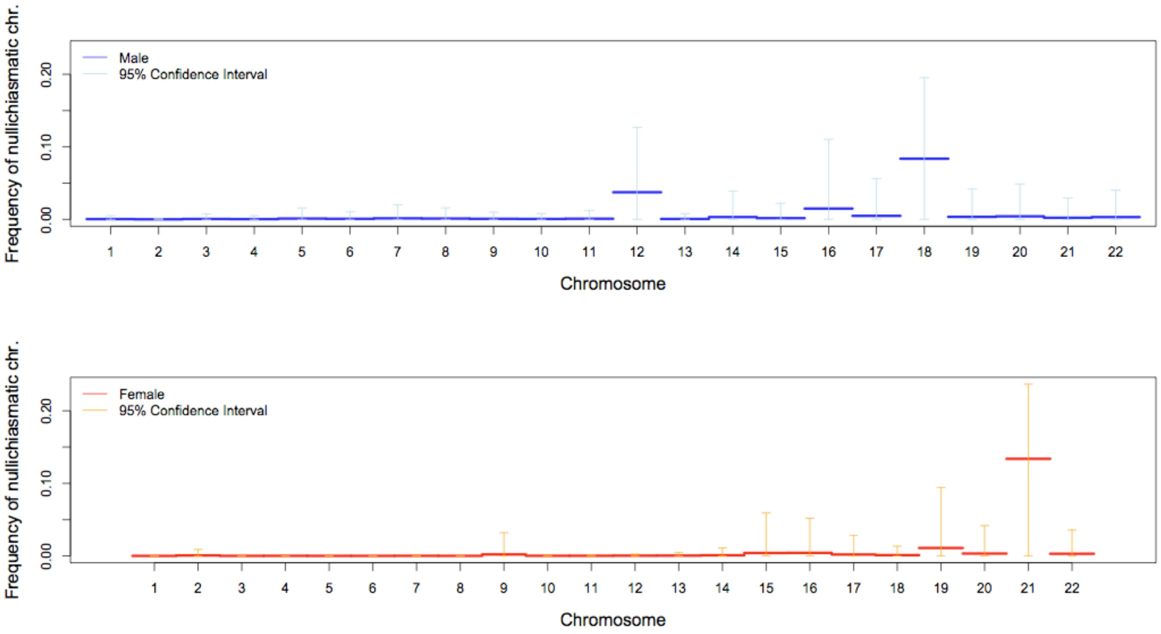

D) For each chromosome, the Bayes Factor in support of a model with nullichiasmatic chromosomes vs. a model with an obligate crossover, for males (blue) and females (red). See the legend of Supplementary Figure 1B for additional details.

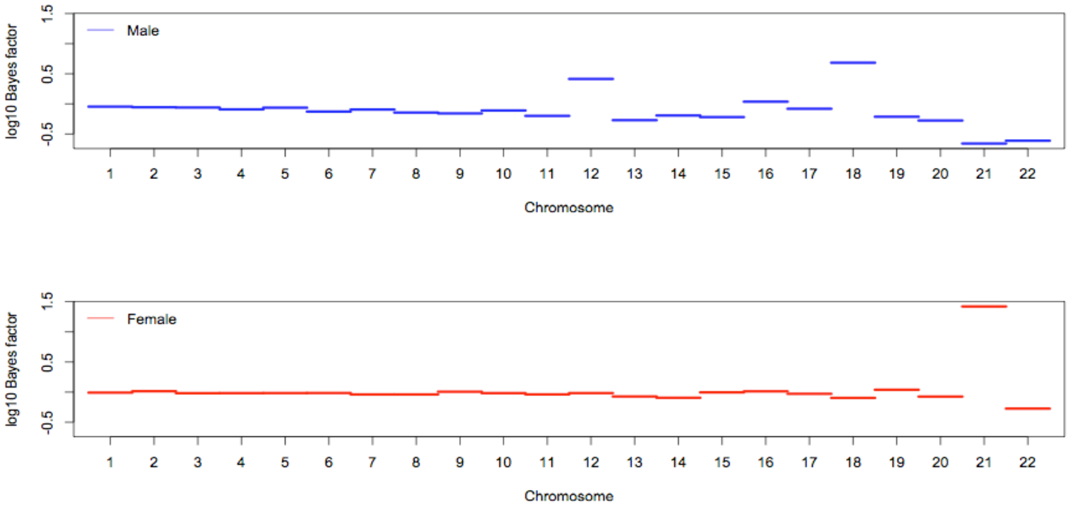
Male
log10 Bayes factor
-0.5
0.5
1.5
1 2 3 4 5 6 7 8 9 10 11 12 13 14 15 16 17 18 19 20 21 22
Chromosome
Female
log10 Bayes factor
-0.5
0.5
1.5
1 2 3 4 5 6 7 8 9 10 11 12 13 14 15 16 17 18 19 20 21 22
Chromosome

Supplementary Figure 2

Sex-specific estimates of the parameter ν from the gamma model, for each chromosome. The intervals correspond to ± twice the estimated standard error. A horizontal line is plotted at the pooled estimate of ν obtained using the data from all autosomes.

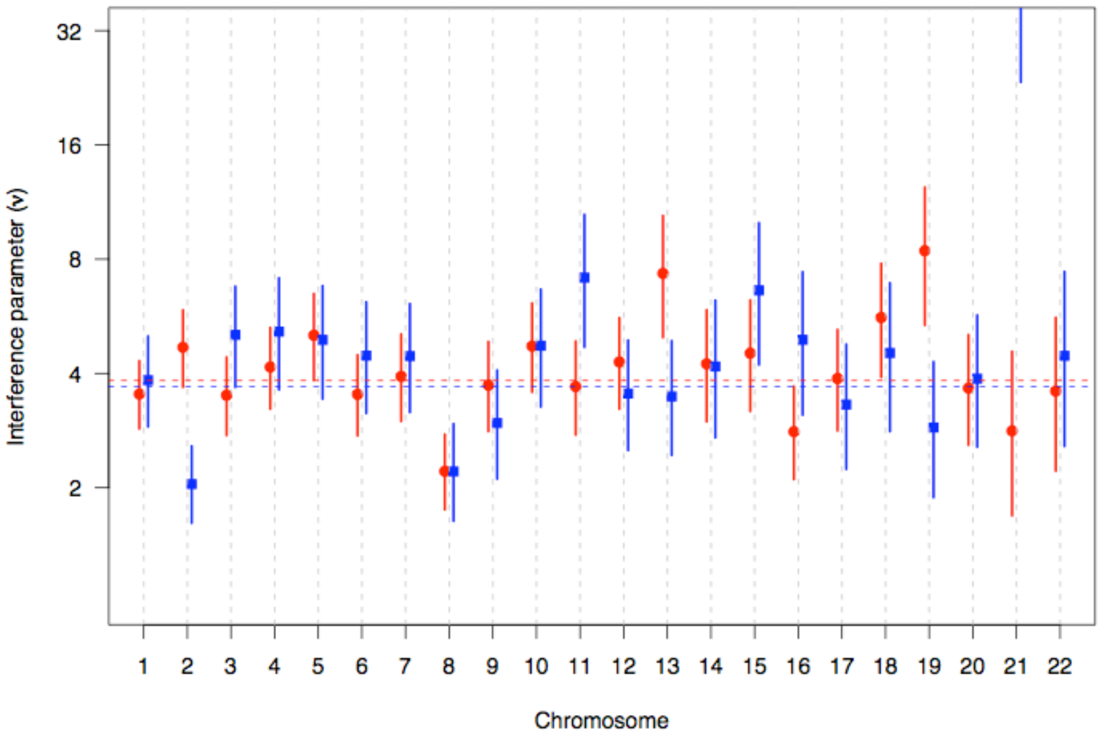

Supplementary Figure 3

Estimated correlation function of the distance from the centromere ρ(d) between recombination events on the p and q arms that both occur within distance d of the centromere. The dashed curves correspond to approximate 95% pointwise confidence limits. The figure was obtained for the Hutterite data, using all meiotic products that had at least one crossover on each arm, pooled across chromosomes, as in Figure 6 of Broman and Weber (2000)[1]. It shows a negative correlation between the positions of recombination events of either side of the centromere, indicating that there is interference across the centromere, i.e., that the centromere is not a barrier to interference.

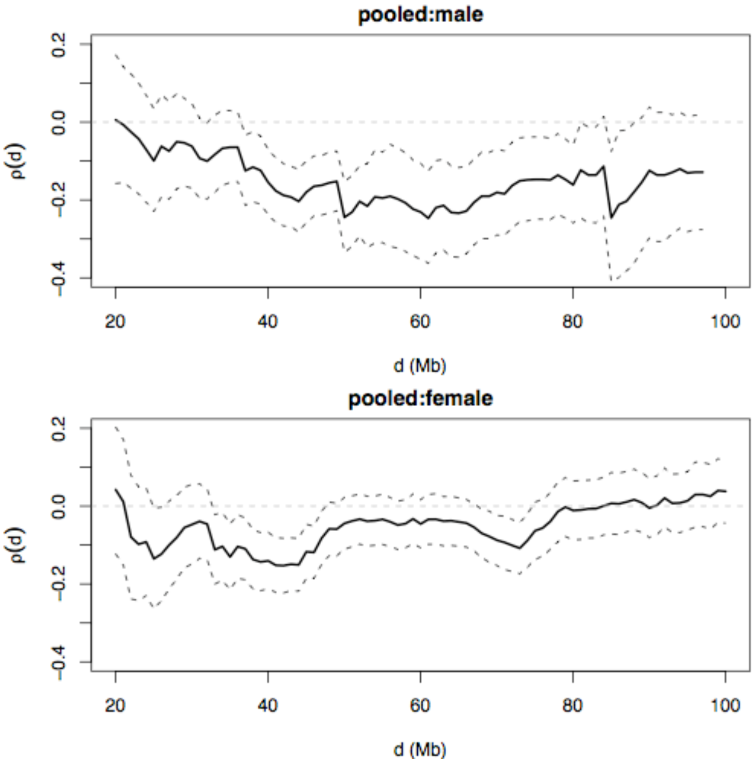

Supplementary Figure 4

Double crossovers in close proximity. The observed number of double crossovers within 5 cM or less *versus* the number expected under a gamma renewal model, the standard statistical model for crossover interference (see SOM). In red are events in female transmissions; in blue in male transmissions.

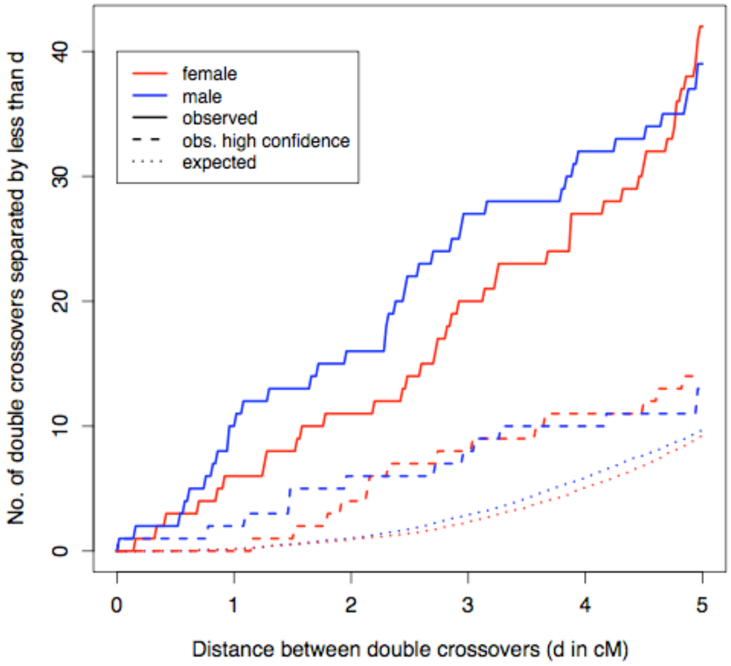

Although the high confidence set was stringently vetted and likely represents an under-estimate of both the number of close double crossovers and their proximity (see Supplementary Methods), there is still an excess number within 5 cM compared to expectation. For example, even if we very conservatively ignored all the crossovers that we excluded because they did not meet one of our vetting criteria, finding so many double crossovers within 5 cM would be unexpected, given the estimated strength of interference (p=0.047 for females and p=0.110 for males, respectively).

We also asked how close we would expect double crossovers to be, given that they occur within 5 cM. When we took the expected relationship shown in this Figure, and used it to simulate 15 events in females and 14 events in males, we found that the mean distance between double crossovers is shorter than expected (one-sided p=0.0039 for females and 0.0028 for males, based on 10,000 simulations).

Supplementary Figure 5

For maternal transmissions of chromosome 21, we rejected a model with no nullichiasmatic tetrads and no chromatid interference (see Figure 2 and main text). To explore whether the data could be explained by chromatid interference alone, we considered a model that mimics its effects, under the assumption that there are no nullichiasmatic tetrads. Of central importance in the model is the parameter k, which controls the variance in the number of crossovers given the number of chiasmata and can thus be viewed as controlling the degree of chromatid interference; $k = 0$ corresponds to the case of no chromatid interference.

The first panel shows the inferred distribution of the number of chiasmata in tetrads under this model. Shown are the point estimates and the 95% confidence interval (see Supplementary Methods for details), for the maximum likelihood estimate $k = 13.26$.

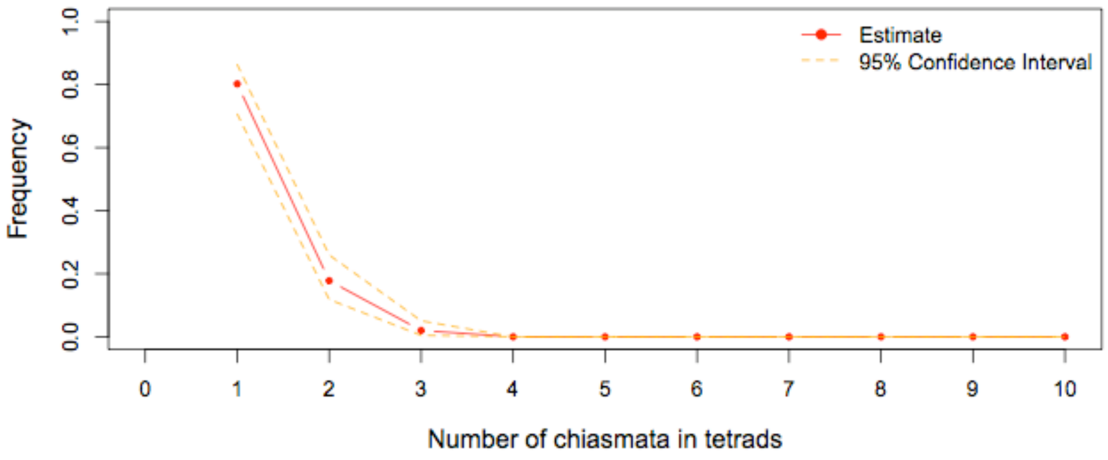

The second panel is the observed distribution of the number of crossovers in female transmissions of chromosome 21 (using our data and those of Ref. [6]), along with the 95% prediction interval (as can be seen, the data fall well within).

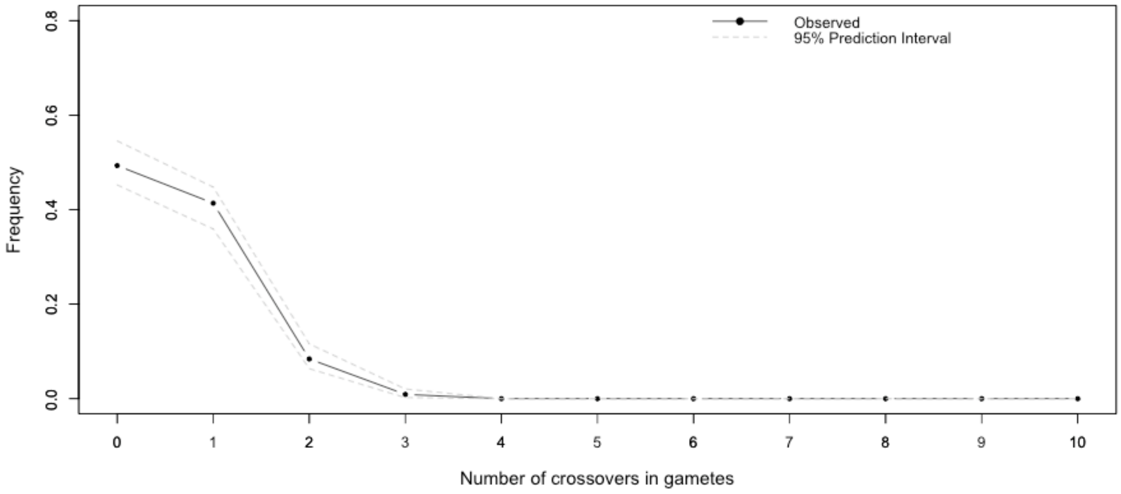

The last panel shows the probability *t* of observing a recombinant gamete given a chiasma, the variance of which is determined by the parameter *k*. Under a model with no chromatid interference, this probability is always 0.5, leading to binomial sampling of recombinant chromatids (see Methods); to mimic chromatid interference, we assumed that *t* follows a symmetric beta distribution, leading to beta-binomial sampling. As can be seen, the estimated distribution of *t* (k = 13.26; shown in red) is extremely variable, suggesting that strong positive chromatid interference would be needed to generate the observed pattern. It is not known if chromatid interference occurs in humans, but none is observed in yeast [14].

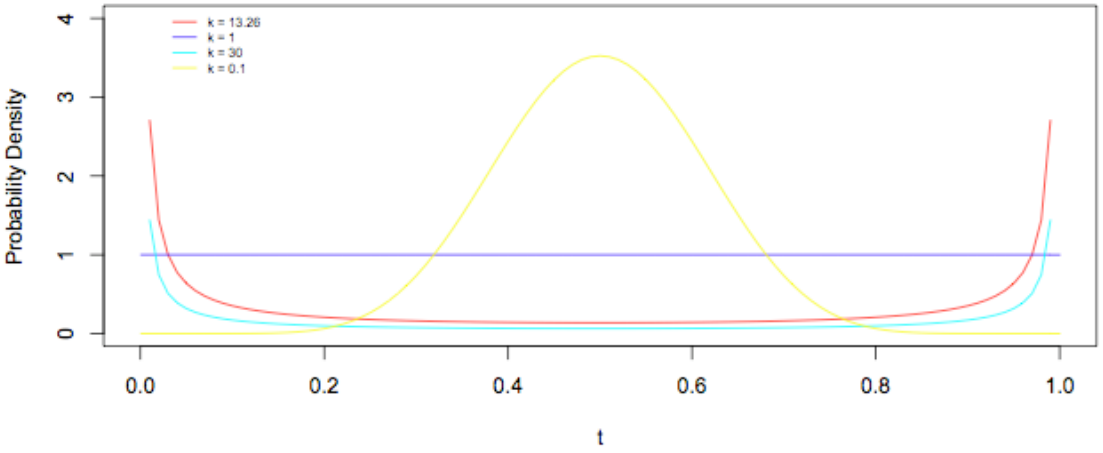

Supplement: Text S1 — Supplementary methods, figures, and tables. (1.14 MB PDF) [file pgen.1000658.s001.pdf]
